# Properties and computational insights of catalysts based on amide linked polymer for photo-Fenton remediation of Rhodamine B dye

**DOI:** 10.1038/s41598-025-13192-z

**Published:** 2025-08-20

**Authors:** Asmaa M Fahim, Khadiga Mohamed Abas

**Affiliations:** 1 Department of Green Chemistry, National Research Centre, P.O. Box.12622, Dokki, Cairo, Egypt; 2 Physical Chemistry Department, Advanced Materials Technology and Mineral Resources Research Institute, National Research Centre, 33 El-Bohouth St, Giza, 12622 Egypt

**Keywords:** Advanced oxidation processes (AOPs), Photo-Fenton catalytic degradation, Amide polymer/Cellulose composite, Bimetallic oxide catalyst, Theortical studies, Reaction kinetics and dynamics, Chemistry, Synthetic chemistry methodology

## Abstract

In this elucidation, the use of advanced oxidation processes (AOPs) is anticipated as a promising green technology for deducting water contamination. Here, we announce the use of an amide polymer accumulated with bimetallic oxide, CuFe_2_O_4_, based on a cellulose moiety as photo-Fenton catalysts. Firstly, the condensation of terephthaloyl dichloride with aminoacetophenone utilized to afford the corresponding N_1_,N_4_-bis(4-acetyl phenyl)terephthalamide (BAT), which easily reacts with carboxymethyl cellulose, resulting in the cleavage of H_2_O and chelation on the cellulose surface, forming the novel N_1_,N_4_-bis(4-acetyl phenyl) terephthalamide/carboxymethyl cellulose (BAT/CMC). It adsorbs bimetallic oxide, CuFe_2_O_4_ through physical interaction to form N1,N4-bis(4-acetylphenyl) terephthalamide/carboxymethyl cellulose/CuFe_2_O_4_ (BAT/CMC/CuFe_2_O_4_). All synthesized compounds were confirmed through spectral analysis, including FT-IR, NMR, SEM, and XRD. In addition to nitrogen adsorption–desorption measurements of evaluated catalysts. Furthermore, the (BAT/CMC/CuFe_2_O_4_) exhibits superior reactivity for Fenton-like reactions in degrading Rhodamine B (RhB) dye under solar irradiation compared to the prepared heterogeneous catalyst, CuFe_2_O_4_. Moreover, under optimal conditions, a comparative experiment between conventional and photo-Fenton catalytic degradation was conducted. After 80 min, BAT/CMC/CuFe_2_O_4_ achieved a maximum removal efficiency for RhB of 39.5% at 303 K, while the photo-Fenton oxidation process completely decomposed RhB (94.2%). The first-order kinetic simulation is the most appropriate model for RhB onto all developed materials, as demonstrated by the higher values of correlation coefficients, R^2^. Thermodynamic studies disclosed that the system functions through endothermic, non-spontaneous processes; also, the created samples have activation energies (Ea) greater than 20 kJ/mol, suggesting a chemical mechanism for RhB decomposition. Four successive cycles were conducted to evaluate the reusability of developed catalysts under optimal conditions, with a drop-in degradation activity. Furhermore, the Density Functional Theory (DFT) investigation of BAT/CMC/CuFe_2_O_4_ with RhB dye using the B3LYP/LANL2DZ(G) basis set confirmed their hydrogen bond interaction and determined their different physical describitors.

**Supplementary Information:**

The online version contains supplementary material available at 10.1038/s41598-025-13192-z.

## Introduction

Water treatment adopting advanced oxidation processes (AOPs) is growing progressively more popular^1,2^. The aforementioned procedures entail treatment at ambient temperature and atmospheric pressure, emphasizing the in situ production of active oxidizing agents, including hydroxyl radicals (HO^•^), in adequate quantities to effectively clean water^3^. Substantial oxidizing and purifying features distinguish H_2_O_2_, the Fenton reagent, which may also transform hazardous organic materials into less dangerous ones^4^. According to the mechanism of HO^•^ creation, there are numerous types of AOPs, such as the conventional Fenton reaction, heterogeneous Fenton-like reaction, and approaches that use combinations of electrical, microwave, ultraviolet, ultrasonic, and so on^5,6^. By ultraviolet (UV)/visible irradiation, Fenton (H_2_O_2_/Fe^2+^) and Fenton-like (H_2_O_2_/Fe^3+^) reactions can be substantially accelerated. Because Fe^2+^ ions are created from Fe^3+^ by photo-reduction, photo-irradiation inhibits the growth of Fe^3+^ ions in the process^7^. Moreover, photo-catalytic oxidation is significantly more favorable, safe, affordable, and efficient^8^. Both a natural or artificial lighting supply in conjunction with photo-catalytic degradable materials are essential for this technique^9,10^. It was assembled from oxides that absorb light, involving metal oxide photo-catalysts, which excite electrons from a lower energy state (VB) to a higher energy state (CB) and yield electron-hole pairs^11^.

The practical applications for monometallic iron catalysts (ZVI, Fe_2_O_3_, Fe_3_O_4_, and Fe(OOH)) are restricted due to their inadequate catalytic efficiency, unstable nature, and lack of recyclability^12^. In contrast, copper-based oxides have emerged as the main focus of research into innovative photo-catalysts because of their benefits: intense light absorption, strong carrier mobility, non-toxicity, sustainability, long-term stability, and low manufacturing costs. According to earlier claims, electron emission across the interface may be sped up by coordination between the redox combinations of iron (Fe^3+^/Fe^2+^) and copper (Cu^2+^/Cu^+^), facilitating the rapid reduction of Fe^3+^^13^. It was proposed that the Fe^2+^ species of the Fe–Cu bimetallic catalyst is mostly maintained by the interaction of Fe^3+^ with Cu^+^, rather than Fe^3+^ being reduced by H_2_O_2_^14^. Consequently, the cohabitation of Fe and Cu on a catalyst’s surface may promote electron transfer in the reaction environment and foster conditions conducive to the emergence of reactive radical species^15^. The reaction always relies on the redox of the metal ions in the single-metal center, regardless of whether the homogeneous or heterogeneous Fenton process is employed.

**Fig. 1:**
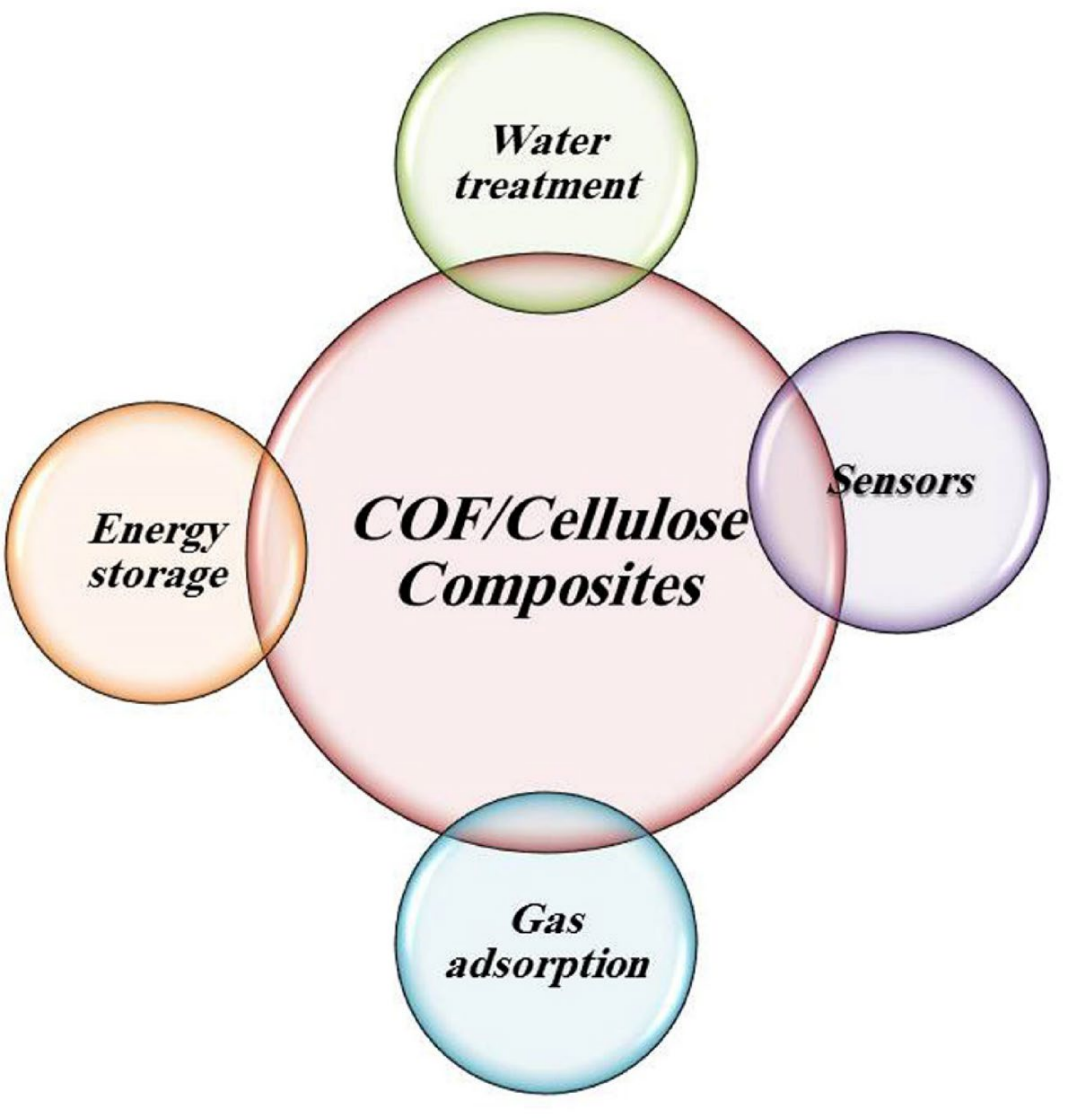
Uses of COF/cellulose composites in different applications.

Fenton catalysts containing metals have a fundamental characteristic that frequently causes issues. These involve the requirement for acidic reaction conditions (pH = 2–4), insufficient H_2_O_2_ consumption^16^, and additional contamination due to the release of metal-containing waste^17^, which restricts the use of Fenton reactions for environmental cleanup. In turn, creating Fenton-like photo-catalysts without metal is considered a reasonable approach to address these drawbacks. To overcome the limitations of the traditional Fenton reaction for environmental cleanup and other applications, double reaction centers (electron-rich and electron-poor centers) are established in a catalyst^18^. H_2_O_2_ does not react directly with the metal species in dual-reaction-center Fenton-like systems; alternatively, it traps extra electrons in electron-rich regions to produce HO^•^. Adjusting band structures and improving catalytic function in metal-free materials require laborious modification procedures such as chemical modifications^19^, elemental manipulation^20^, and heterojunction creation^21,22^. Over the past 10 years, covalent organic frameworks (COFs) have garnered significant interest. With their substantial porosity, tunable structures, low density, and outstanding thermal and chemical stability^23–25^, Covalent organic framework (COF) entirely of organic building blocks with reversible covalent bonds have an extensive number of prospective applications, including radioactive iodine adsorption^26,27^, energy storage^28^, and photo-catalysis^29^. Furthermore, they have benefits like high stability, adjustable porosity, and useful design. Moreover, COFs are used in different applications such as water treatment and waste minimization. Because of cellulose’s plentiful supply, sustainable nature, and adaptable chemistry, research into incorporating cellulose into COFs has just recently begun^30,31^. Cellulose is one of the most prevalent biopolymers on the planet. It can improve structural integrity, environmental friendliness, and certain functionality when incorporated into COFs and used in many applications as displayed in Fig. 1.

Among the many industries with a wide range of sectors and an intricate industrial chain is the textile industry. The primary issue with the textile industry’s detrimental effect on the environment is wastewater release. The majority of water contamination is triggered by finishing and dyeing operations^32^. Hazardous substances harmful to the environment and people’s health have been identified in the dyeing industry’s waste byproducts^33^. An artificial cationic dye with a multi-ring aromatic xanthene core planar structure is referred to as Rhodamine B (RhB) dye^34^. It is often exploited in printing and dyeing processes^35^. The well-documented carcinogenic, mutagenic, and toxic properties of RhB necessitate treating RhB-contaminated discharges before eliminating them^36–38^. RhB has been remediated from water employing traditional approaches such as adsorption^39,40^, biodegradation^41^, micro-wave catalysis^42^, chemical oxidation^33^, and nano-filtration^43,44^. However, the primary criticisms of these processes are their costly nature, lengthy process time, excessive energy use, challenges with regeneration, and pollutants transferring from one step to another. Considerable work has gone into developing substitute remediation techniques that can successfully purify waterways tainted by artificial chemicals^45^.

In this study, an amide polymer forming a covalent organic framework was synthesized through the formation of an amide linkage by condensing acid chloride and aminoacetophenone to produce (BAT), which can easily react with cellulose to afford (BAT/CMC) and chelate with the bimetallic oxide CuFe_2_O_4_ through physical interaction. They were characterized using FT-IR, NMR, SEM, and XRD. Toxic RhB dye was degraded from liquid solutions using a photo-Fenton catalytic degradation approach with the best prepared catalysts and standardized with a bimetallic catalyst of CuFe_2_O_4_. Fenton conditions, including oxidant dosage, pH, dye concentration, and contact time in the presence of solar light, have been optimized. The maximum degradation efficiency using photo-Fenton catalytic oxidation of RhB dye has been compared with the maximum degradation efficiency using the classical Fenton process in dark. Kinetic and thermodynamic studies of photo-Fenton reactions have been modeled. Moreover, the synthezied polymer compounds decomposed on the surface of cellulose in the presence of bimetallic oxide and reacted with Rhodamine B via chemical interaction with the external COO^−^, enhancing its color change. These results were obtained from the optimization of these compounds through DFT/B3LYP/LANL2DZ(G) basis set.

## Materials and methods

### Instruments and techniques

The Shimadzu FT-IR 8101 PC infrared spectrophotometer recorded the Fourier Transform Infrared (FTIR) spectra. The ^1^H-NMR and ^13^C-NMR spectra were determined in DMSO-d_6_ at 300 MHz on a Varian Mercury VX 300 NMR spectrometer (^1^H at 300 MHz) exhausting trimethyl silane as an internal typical. Scanning electron microscopy (SEM) was investigated utilizing JEOL JXA-840 A electron probe Micro-analyzer and were air-dried before imaging, and images were obtained using an accelerating voltage of 10–15 kV. The X-ray diffraction (XRD) patterns were investigated using a Diano X-ray diffractometer with a CuKα radiation source energized at 45 kV and a Philips X-ray diffractometer (PW 1930 generator, PW 1820 goniometer) with CuK radiation source (λ = 0.15418 nm), at a diffraction angle range of 2θ from 10 to 70 °C in reflection mode. Adsorption–desorption of N_2_ at − 196 °C was performed to assess the pore properties of the synthesized samples and the specific surface area using the Brunauer–Emmett–Teller (BET) approach with a Quanta Chrome Instruments NOVA Automated Gas Sorption System, Version 1.12, USA.

The point of zero charge (pH_PZC_) of the evaluated catalysts was ascertained by diluting 0.3 g of the samples with 20 ml of pH-adjusted solutions varying from 2 to 12, then shaking the solutions on a settled shaker for 24 h. Following shaking, the final pH (pH_f_) was recorded, and ΔpH was plotted against the initial pH (pH_i_). Solutions of 0.1 M HCl and 0.1 M NaOH were used to adjust the pH.

### Chemicals and reagents

Terephthaloyl dichloride, 1-(4-aminophenyl)ethan-1-one, and dioxane were purchased from Aldrich Chemical Co. Carboxy methyl cellulose (CMC) was purchased from Rasyan laboratory. Cupric chloride (CuCl_2_⋅2H_2_O) and other reagents like ferric chloride hexahydrate (FeCl_3_·6H_2_O) and ethanol were of analytical grade, purchased from Sigma Aldrich (Shanghai, China). HCl (35%) and NaOH were supplied by Merck Chemicals Co. Ltd. Hydrogen peroxide (H_2_O_2_, 30%) was purchased from Fisher Scientific Co. Rhodamine B dye (RhB) dye was acquired from Fisher Scientific Co.

### Formation of N_1_,N_4_-bis(4-acetylphenyl)terephthalamide (BAT)

Condensation between terephthaloyl dichloride^46^ (2.5, 10 mmol) and 1-(4-aminophenyl)ethan-1-one^47,48^ (1.35, 10 mmol) was carried out in the presence of acetic acid as an acidic catalyst (0.1 ml, 4 M) in a dioxane solution. The reaction mixture was stirred at room temperature, forming a pale purple solid which was filtered off, crystallized with EtOH/ dioxane, dried under vacuum for 24 h, and weighed to yield 82%.

### Reactivity of CMC with N_1_,N_4_-bis(4-acetylphenyl) terephthalamide (BAT)

The N_1_, N_4_-bis(4-acetyl phenyl)terephthalamide (2.1 g, 5 mmol) was mixed with CMC^49^ (2.1 g, 10 mmol) and stirred at 50 °C for 2 h in dioxane. It was then filtered, dried, and washed several times with EtOH/dioxane, yielding 75%.

### Physical interaction of BAT/CMC with CuFe_2_O_4_ nanoparticles

Using hydrothermal and sonochemical techniques in a basic medium, the CuFe_2_O_4_ nanoparticles were created (materials and methods described in SI). Also, the formed BAT/CMC (1 g) in 25 ml distilled water was stirred with a solution of CuFe_2_O_4_ (0.25 g) in dioxane for 5 h to get the BAT/CMC/CuFe_2_O_4_^50^.

### Photo-Fenton catalytic degradation setup

The RhB stock solution (0.5 g/L) was diluted to the necessary concentrations (20–100 mg/L) and preserved in a brown reagent container to avoid dye decomposition unless stated otherwise. In batch studies, synthesized compounds (1 g/L) were added to the RhB dye solution, and the reaction mixture was conducted at 303 K under sunlight. In a traditional experimental setup, the dye solution was incorporated with the relevant amounts of BAT, CuFe_2_O_4_, and BAT/CMC/CuFe_2_O_4_ in open glass reactors. An 0.1 M HCl and 0.1 M NaOH were added to adjust the solutions’ pH to the appropriate value. Normally, the prepared samples and H_2_O_2_ (30–120 mM) were added while vigorously agitating the RhB solution at the intended starting pH value (2–6). Sunny days were used for solar testing. Two-milliliter aliquots were taken at predetermined intervals, and the supernatant was extracted for UV-Vis inspection by centrifuging the samples periodically. Using a UV-2401PC spectrophotometer, the maximum absorption wavelength (λ_max_) of the aqueous solutions’ UV-Vis absorption spectra, λ_max_ of 550 nm, was detected. The temperature of the solution was monitored by a thermostatic water bath to explore the impact of temperature on the rate of RhB degradation. To analyze the conventional Fenton catalytic degradation of RhB dye in the absence of sunlight, the reactor was operated in closed black bottles to preclude possible photochemical reactions. The following equation was applied to estimate the photo-Fenton degradation efficiency to optimize the reaction terms:

1 $$\varvec{P}\varvec{h}\varvec{o}\varvec{t}\varvec{o}\varvec{d}\varvec{e}\varvec{g}\varvec{r}\varvec{a}\varvec{d}\varvec{a}\varvec{t}\varvec{i}\varvec{o}\varvec{n}\left(\varvec{\%}\right)=\left(\frac{{\varvec{C}}_{^\circ}-{\varvec{C}}_{\varvec{t}}}{{\varvec{C}}_{^\circ}}\right).100$$

The dye’s absorbance before and after the Fenton reaction (at time t) is represented by the symbols C_°_ and C_t_. At consistent intervals of ten minutes, reactant solutions containing RhB (40 mg/L) and optimal reaction setups at three different temperatures (303, 313, and 323 K) were periodically surveyed to gauge the kinetic and thermodynamic characteristics of Fenton oxidation processes.

### Fenton kinetic studies

Implementing the pseudo-first and pseudo-second order equations (Eqs. 2 and 3), respectively at various temperatures (303, 313, and 323 K) for BAT, CuFe_2_O_4_, and BAT/CMC/CuFe_2_O_4_, a kinetic study of RhB dye degradation was conducted.

2 $$\varvec{ln}\left(\frac{{\varvec{C}}_{}}{{\varvec{C}}_{^\circ}}\right)=-{\varvec{K}}_{\varvec{a}\varvec{p}\varvec{p}}.\varvec{t}$$

K_app_ (min^− 1^) reflects the apparent value of the first-order rate constant for the organic target compound decomposition. The second-order reaction’s rate law may be formulated with the following equation:

3 $$\frac{1}{\left[\varvec{C}\right]}={\varvec{K}}_{2}\varvec{t}+\frac{1}{\left[{\varvec{C}}_{^\circ}\right]}$$

Where K_2_ depicts the rate constant of the pseudo-second-order equation (min^− 1^).

### Fenton thermodynamic studies

The study assessed the impact of different temperatures, specifically 303, 313, and 323 K, on the photo-Fenton degradation of RhB dye using developed catalysts. Utilizing the Arrhenius relation, the fluctuation of the apparent first-order rate constant, K_1_, with temperature was applied to determine the activation energy (Ea) and various thermodynamic parameters^51.^

4 $${\varvec{K}}_{\varvec{a}\varvec{p}\varvec{p}}=\varvec{A}.\varvec{exp}\left(-\frac{{\varvec{E}}_{\varvec{a}}}{\varvec{R}.\varvec{T}}\right)$$

Where A defines the pre-exponential factor (or frequency; min^− 1^), Ea represents the apparent activation energy (k.J.mol^− 1^), R refers to the universal gas constant (8.314 J.mol^− 1^. K^− 1^), and T is the absolute temperature (K). Equation (4) can be illustrated in its linearized form as Eq. (5).

5 $$\varvec{l}\varvec{n}{\varvec{K}}_{\varvec{a}\varvec{p}\varvec{p}}=-\frac{{\varvec{E}}_{\varvec{a}}}{\varvec{R}\varvec{T}}+\varvec{l}\varvec{n}\varvec{A}$$

Using the information obtained from kinetic modeling, the Eyring-Polanyi equation (Eq. 6) was utilized to ascertain the thermodynamic attributes.

6 $$\varvec{ln}\left(\frac{\varvec{K}}{\varvec{T}}\right)=-\frac{\varvec{\varDelta}{\varvec{H}}^{\varvec{*}}}{\varvec{R}}.\frac{1}{\varvec{T}}+\varvec{ln}\left(\frac{{\varvec{K}}_{\varvec{B}}}{\varvec{h}}\right)+\frac{\varvec{\varDelta}{\varvec{S}}^{\varvec{*}}}{\varvec{R}}$$

Where K_B_ presents the Boltzmann constant 1.3806 × 10^− 23^ m^2^.kg.min^− 2^.K^− 1^, ΔH* and ΔS* serve as the enthalpy (kJ/mol) and entropy (kJ/mol.K), respectively, h implies the Blank constant 6.626 × 10^–34^ m^2^.kg/min. Applying the values of ΔS* and ΔH* in the Eq. 7, the value of ΔG*, Gibbs free energy (kJ/mol), can be estimated^52^.

7 $$\varvec{\varDelta}{\varvec{G}}^{\varvec{*}}=\varvec{\varDelta}{\varvec{H}}^{\varvec{*}}-\varvec{T}.\varvec{\varDelta}{\varvec{S}}^{\varvec{*}}$$

## Results and discussion

### Synthesis of amide polymer (N_1_,N_4_-bis(4-acetylphenyl)terephthalamide (BAT))

**Fig. 2:**
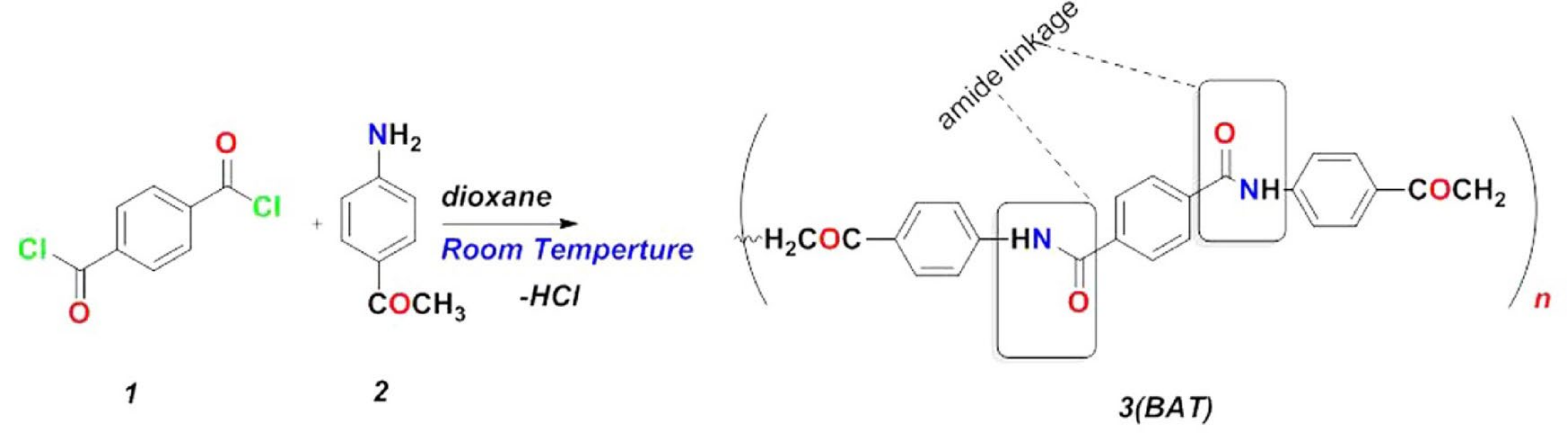
Condensation of terephthaloyl dichloride (1) with 1-(4-aminophenyl)ethan-1-one (2).

The nucleophilic addition reaction of terephthaloyl dichloride^46^ with 1-(4-aminophenyl)ethan-1-one in dioxane, stirred at room temperature, results in the elimination of –HCl and affords the corresponding N_1_, N_4_-bis(4-acetylphenyl) terephthalamide. This compound acts as a covalent organic framework, elucidated with spectral investigation such as ^1^HNMR analysis and showed the presence of phenyl rings at 7.9 to 8.1 ppm with multiple protons and the -NH group at the 10.45 ppm, as displayed in Fig. 2.

### Reactivity of BAT polymer with CMC and its interaction with bimetallic oxide (CuFe_2_O_4_)

**Fig. 3:**
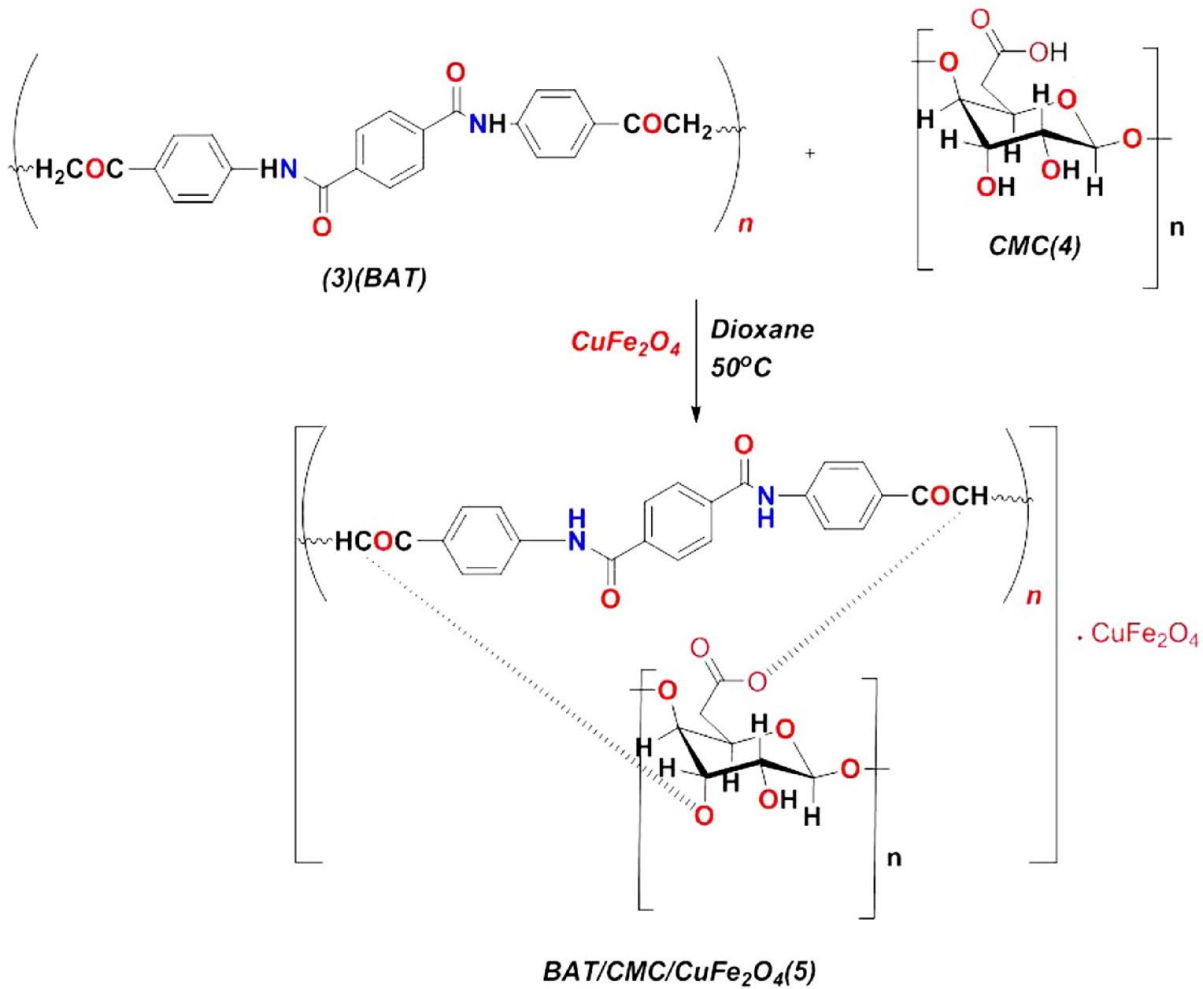
Reaction mechanism of BAT polymer (3) with CMC (4) for BAT/CMC/CuFe_2_O_4_ (5) formation.

The reactivity of CMC^49^ with BAT^53^ polymer was studied by condensing them in the presence of dioxane at 50 °C for 2 h. This process involved the cleavage of two molecules of H_2_O, resulting in chelation on the surface of cellulose and forming novel BAT/CMC. The product was filtered and recrystallized using an EtOH/dioxane mixture. It can interact with the bimetallic oxide, CuFe_2_O_4_ nanoparticles, while coating on the surface of BAT/CMC in dioxane at 50 °C. Their physical interactions are displayed in Fig. 3. and were investigated using spectral analysis.

### FT-IR investigation

**Fig. 4:**
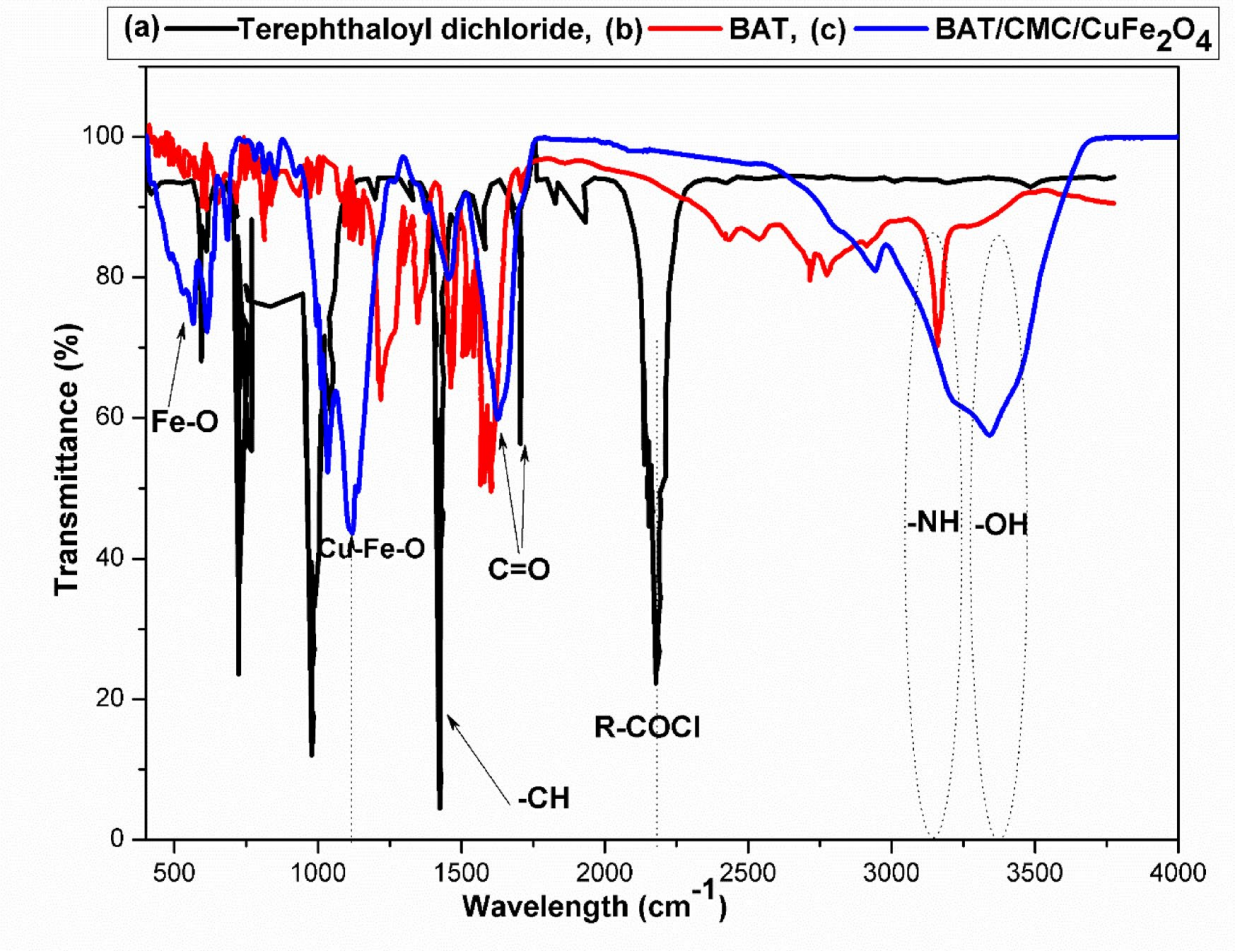
FT-IR spectra of; (**a**) Terphthaloyl chloride, (**b**) BAT, and (**c**) BAT/CMC/CuFe_2_O_4_.

FT-IR spectroscopy revealed the successful synthesis of the novel amide-linkage polymer, N_1_, N_4_-bis(4-acetyl phenyl)terephthalamide (BAT), which showed distinct absorption peaks, as demonstrated in Fig. 4. Firstly, Fig. 4a of terephthaloyl dichloride exhibited no absorption peaks in the range of 3000–3500 cm^− 1^. A very strong C = O stretching vibration of acid chloride appeared at 1740–1780 cm^− 1^ (RCOCl), and the aromatic C = C stretching showed at 1600 cm^− 1^^53,54^. Moreover, the formation of N_1_, N_4_-bis(4-acetylphenyl)terephthalamide (BAT) showed a main peak of overlapping -NH stretching at 3540 cm^− 1^, due to the formation of an amide linkage. The absence of RCOCl and the formation of a new amide C = O band at 1640–1648 cm^− 1^ confirmed the conversion of hydrazide linkage (CONH). The aromatic C = C stretching vibarion appeared at 1580–1600 cm^− 1^, and different absorption bands appeared at 1487, 1376 and 1298 cm^− 1^; respectively, indicating CH_2_ bending vibration in phenyl rings and benzene core, as noticeable from Fig. 4b.

Figure 4c presents the FT-IR analysis of BAT addition to CMC, showing the presence of OH group stretching vibration in the glucose unit at 3432 cm^− 1^. The NH stretching vibration also appeared with OH in the same region at 3326 cm^− 1^, and high stretching vibrations due to strong hydrogen bonding. Additionally, a CH bending vibration of phenyl protons was observed at 1259 cm^− 1^. The vibrational assignments for the bimetallic oxide CuFe_2_O_4_ nanoparticles added to the BAT/CMC surface showed Fe-O group at 3405 cm^− 1^, with bending signals at 622 and 570 cm^− 1^. The C = O of the BAT/CMC/CuFe_2_O_4_ appeared at 1654 cm^− 1^. The Cu-Fe-O showed sharp intensity signals at 1110 and 1039 cm^− 1^, with Cu-Fe-O stretching at 900 cm^− 1^, confirming the incorporation of CuFe_2_O_4_ nanoparticles into the polymer matrix. Thus, we deduced that the presence of CMC expanded the region of the hydroxyl group enclosed by the CONH bond. The involvement of CuFe_2_O_4_ on the surface suggested a physical interaction with Cu-Fe-O in the bending region, located around the BAT/CMC as depicted in Fig. 4c.

### NMR analysis

**Fig. 5:**
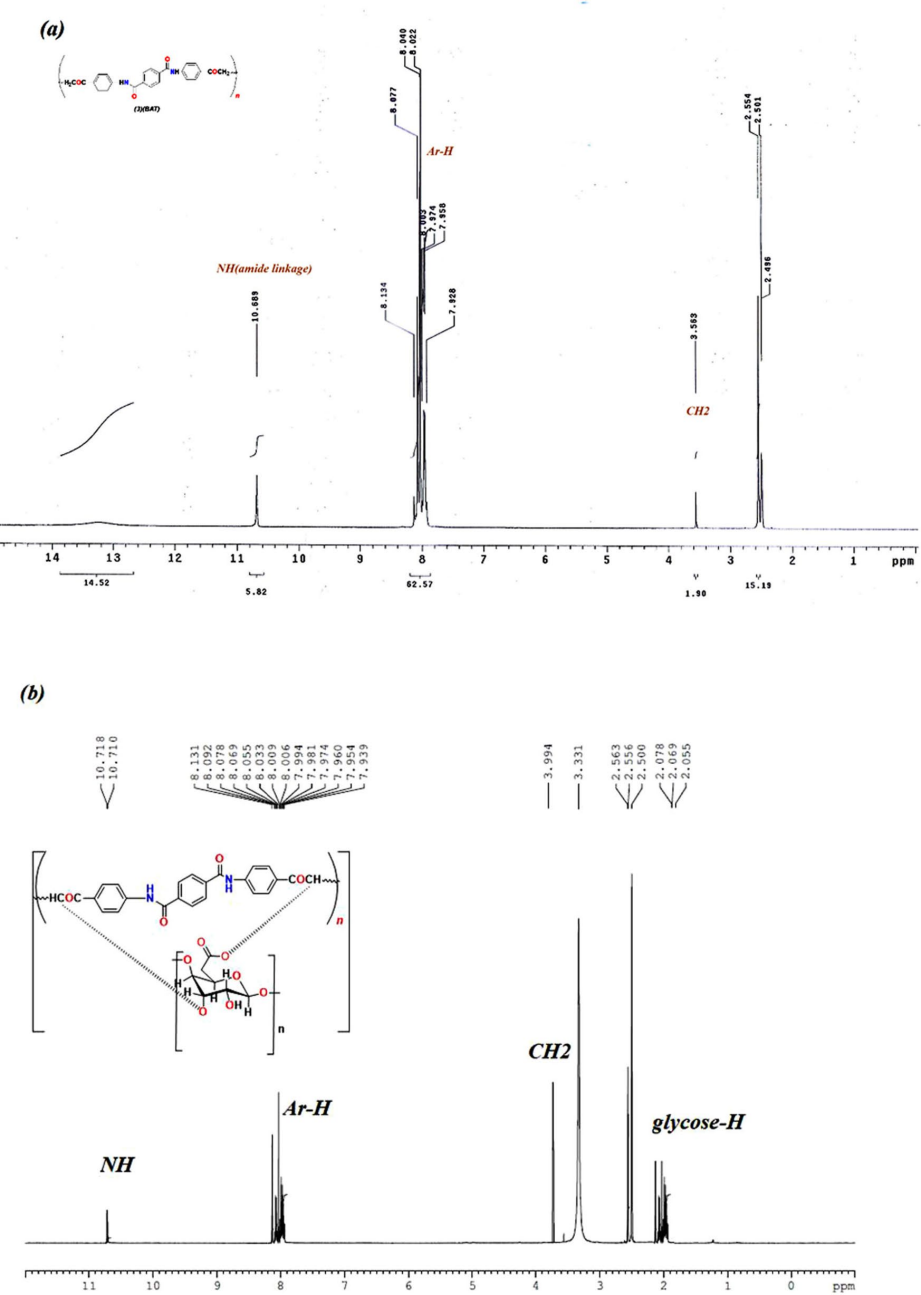
NMR analysis of; (**a**) BAT, and (**b**) BAT/CMC.

The NMR analysis of BAT showed the presence of multiple Ar-H protons in the range of 7.9–8.13 ppm, and the NH signal appeared at 10.68 ppm, as shown in Fig. 5a. Moreover, the NMR analysis of BAT/CMC displayed glucose unit signals as multiple protons at 2.05–2.07 ppm, CH at 3.99 pm, and phenyl protons at 7.9–8.1 ppm. The NH nature in both BAT and BAT/CMC exhibits a similar signal adjacent to C = O. In the BAT polymer, the NH is involved in intramolecular hydrogen bonding with its carbonyl. While the BAT/CMC composite shows additional H-bonding to –COO^−^ and OH groups, which shifts the signal further downfield. This confirms the interaction between BAT and CMC from the external protons, as shown in Fig. 5b.

### XRD analysis

**Fig. 6:**
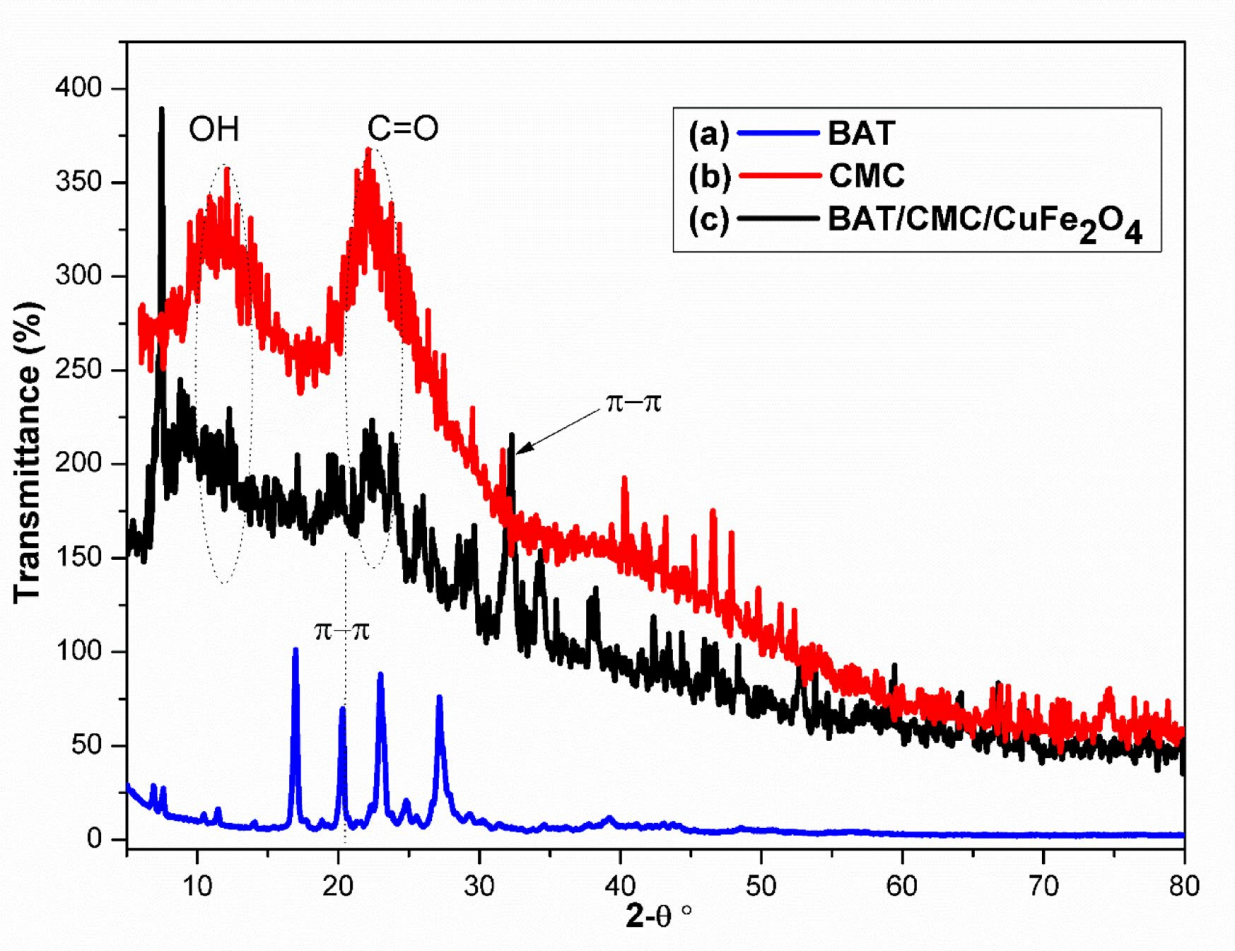
XRD spectral characterization of; (**a**) BAT, (**b**) CMC, and (**c**) BAT/CMC/CuFe_2_O_4_.

Powder X-ray diffraction (PXRD) measurements of BAT (blue) revealed significant peaks, exhibiting sharp, crystalline reflections at 2θ ≃ 7°, 20°, 24°, and 28°. These are characteristic to the (001), (110), (111), and (200) planes. A low-intensity peak at 2θ ~ 8.2° was also observed, coinciding with the (100) plane, likely due to π–π stacking between BAT layers. The d-spacing for BAT was determined to be 3.19 Å, suggesting stronger contact and distinct diffraction peaks (Fig. 6a). The XRD of the CMC (red) showed an amorphous structure with two large “humps” (circled) centred at 2θ ≃ 11.1° and ≃ 22.6°, attributed to hydroxyl and C = O groups within the cellulose backbone (Fig. 6b). The BAT/CMC/CuFe_2_O_4_ composite (black) combines features of both the broad CMC bands at 2θ ≃ 11.1° and ≃ 22.6° and a new, sharp feature at 2θ ≃ 33° attributed to either the spinel CuFe_2_O_4_ phase or π–π stacking interactions between conjugated BAT domains and cellulose surface. These interactions altered the cellulose surface, introducing CuFe_2_O_4_ at 2θ ≃ 8.03°, decreasing cellulose intensity at 2θ ≃ 22.6°, and interacting with the cellulose surface at 2θ = 32.54° (311), 43° (400), and 57° (511), as shown in Fig. 6c and Scherrer’s Eq. 

8 $$\user2{D} = \frac{{\user2{K\lambda }}}{{\user2{\beta Cos}\theta}}$$

K is the shape factor (typically 0.9), λ is the X-ray wavelength (e.g. CuKα = 1.5406 Å), β is the full-width at half-maximum (FWHM, in radians) of the chosen diffraction peak, and θ is the Bragg angle of that peak. For CuFe_2_O_4_, the (311) reflection near 2θ ≃ 35.5°, the (400) peak at ≃ 43°, and the (511) at ≃ 62° are ideal for calculation.

### SEM investigation

**Fig. 7:**
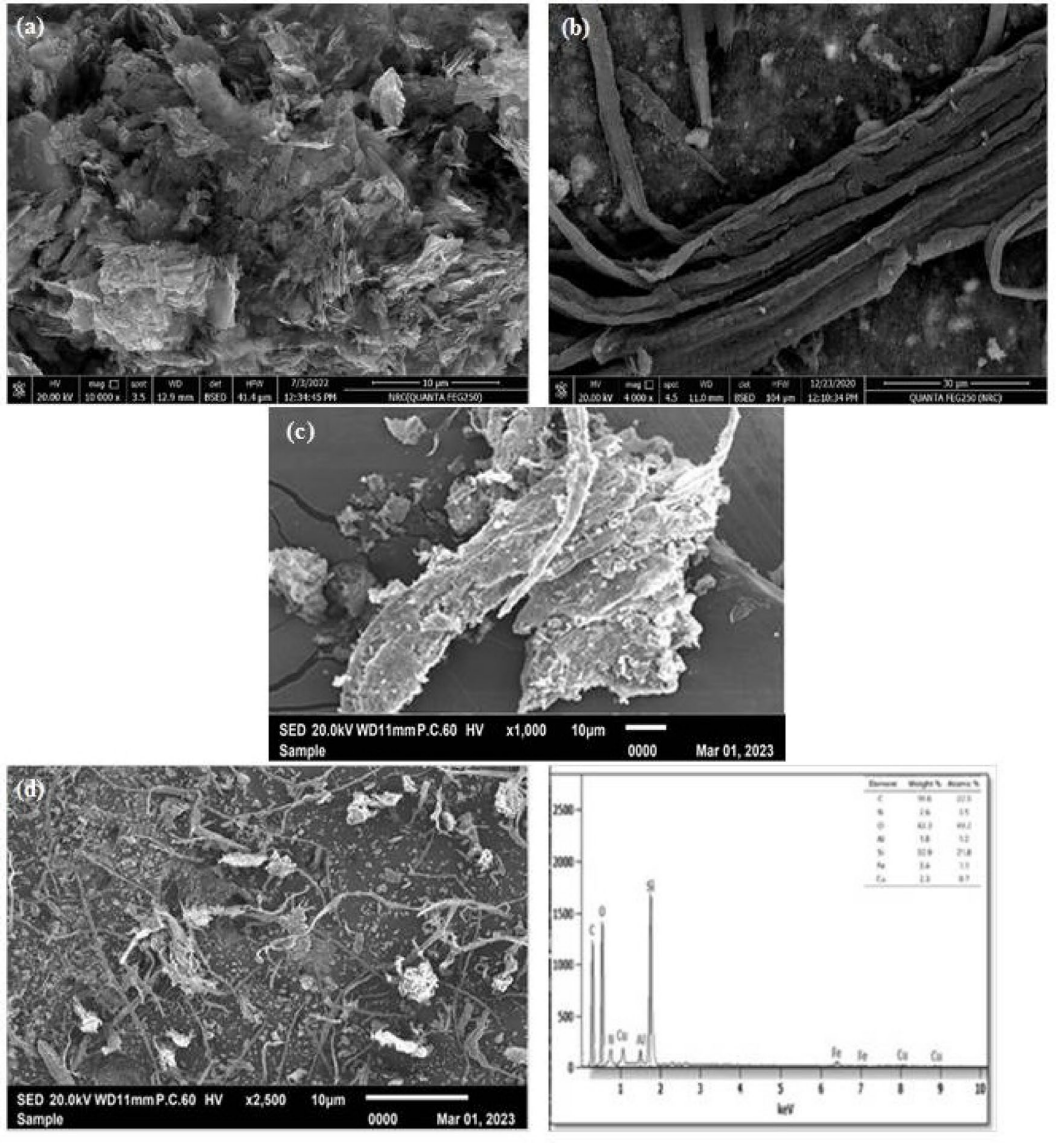
SEM analysis of; (**a**) BAT, (**b**) CMC, (**c**) BAT/CMC, and (**d**) BAT/CMC/CuFe_2_O_4_.

The scanning electron microscope investigated the surface of BAT, CMC, BAT/CMC, and BAT/CMC/CuFe_2_O_4_. Their particle sizes varied significantly, with a mean of approximately 0.54 μm. First, BAT’s surface morphology revealed a dense network of irregular, plate-like flakes piled and interlocked in a three-dimensional, fish-scale-like architecture. Each flake was about 1–5 μm broad. The plates’ jagged surfaces showed that a multilayer structure had been mechanically cleaved or exfoliated, which suggested a high surface area (Fig. 7a). Additionally, a network of long, ribbon-like sheets spread across a reasonably rough substrate is visible in the SEM of CMC (Fig. 7b). Each “ribbon” looks like a stack of thin lamellae that are a few microns wide, 5 to 15 μm long, and have irregular, slightly wavy edges. Many sheets are folded or partially overlapped, highlighting geographic relief with contrast fluctuations and darkened areas. Sub-micron granular debris punctuates the background beneath the ribbons, indicating substrate roughness or particle remnants. Rather than distinct, equiaxed flakes, the overall appearance suggests a layered, plate-like substance exfoliated or sliced into long, flexible strips. Additionally, the BAT/CMC interaction (Fig. 7c) revealed asymmetric, plate-like cluster pieces that seem to be peeled or cleft layers of the prepared material. Each flake is roughly 5–15 μm long and 1–3 μm wide, with a rough, particulate-covered surface and extremely uneven, ripped edges. To highlight their three-dimensional relief, some sheets overlap and arch, producing shadowy areas. The background is a comparatively smooth substrate broken up by tiny clusters of detritus (sub-micron nodules), which could be adsorbates or leftover particles. Figure 7d shows a lamellar, loosely packed morphology with noticeable surface roughness and signs of mechanical exfoliation. CuFe_2_O_4_ was added to the BAT/CMC surface, revealing a three-dimensional, chaotic mat of thin, ribbon-like lamellae scattered with finer granular detritus. With several platelets coiled, folded, or overlapped, the lamellae are roughly 5–20 μm long and 1–3 μm wide, creating noticeable topographic contrast and shadowing. Sub-micron particles that stick to or rest between the bigger sheets punctuate the background substrate, which appears quite smooth. EDX revealed distinct percentages of C (14.6%), N (2.6%), O (42.3%), Fe (3.4%), and Cu (2.3%), confirming accumulation of CuFe_2_O_4_ on the BAT/CMC surface, as displayed in Fig. 7d. This confirms their physical interaction, as seen in Fig. 3.

## Photo-Fenton catalytic degradation of RhB dye

Regarding the superior functionalization findings of prepared amide linkage polymer-based compounds, BAT and BAT/CMC/CuFe_2_O_4_ were selected as photo-Fenton catalysts to study their oxidative degradation efficiency and mechanisms towards RhB dye comparatively to the standard bimetallic catalyst CuFe_2_O_4_.

### Determination for point of zero charge

By identifying the sort of surface charge and determining the pH at the point of zero charge (pH_pzc_), one can assess the amount of accessible active sites a catalyst possesses, which is influenced by changes in the medium pH. When the pH decreased below pH_pzc_, the surface of the developed samples exhibits protonated functionalities at higher pH values above pH_pzc_, competition between anionic species and OH^−^ ions in solution reduces the removal capacity^55^.

The pH at zero point charge (pH_pzc_) for BAT, CuFe_2_O_4_, and BAT/CMC/CuFe_2_O_4_ were evaluated to be 3.5, 6.6, and 6.7, respectively as figured out in Fig. S1 (Supporting Information). The prepared samples’ surfaces exhibit a protonated functionality for BAT, neutral surfaces for CuFe_2_O_4_ and BAT/CMC/CuFe_2_O_4_^56^. Thus, the obtained BAT/CMC/CuFe_2_O_4_, and CuFe_2_O_4_ are more attractive to cationic charged particles^57^.

### Controlling parameters

**Fig. 8:**
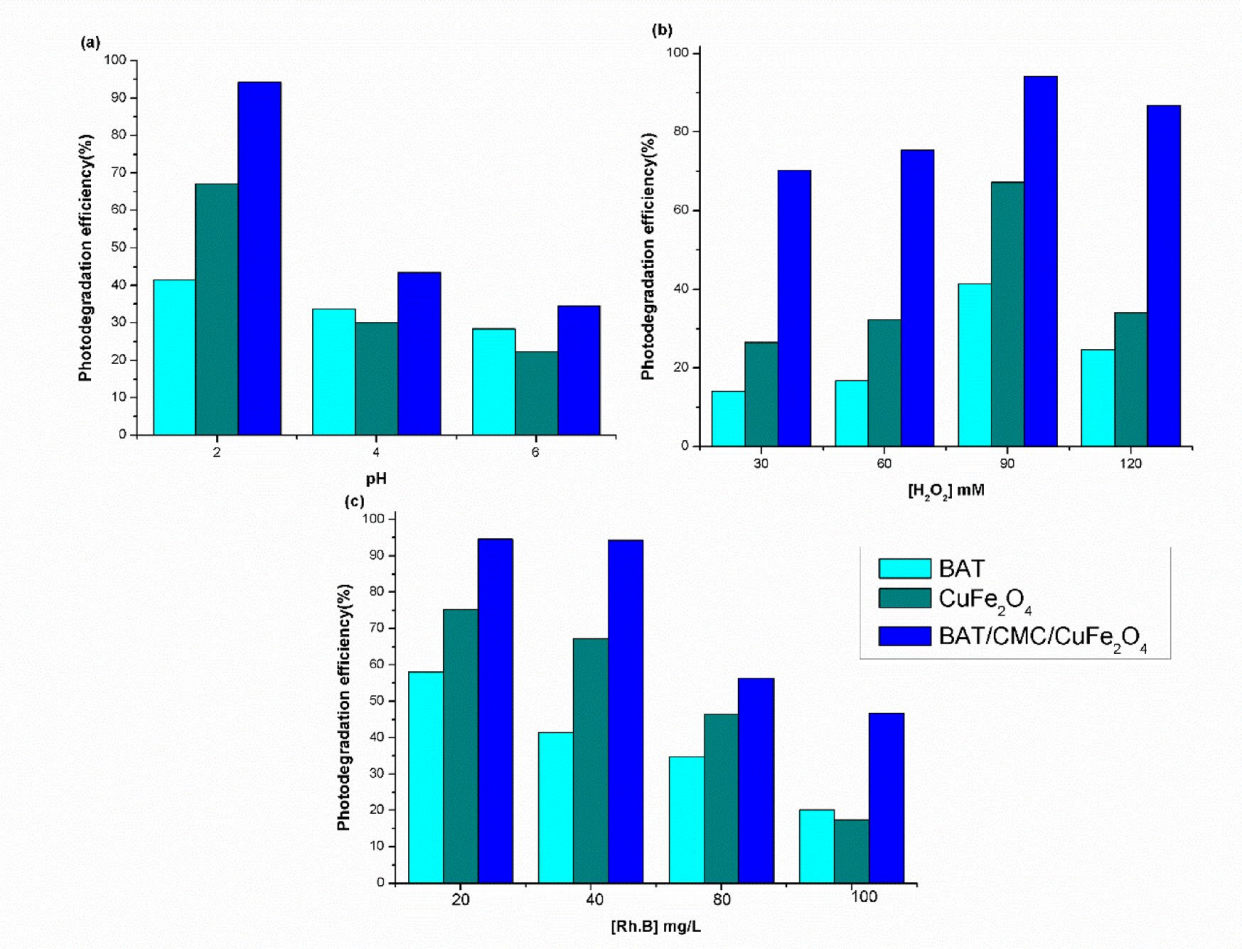
Effect of operating conditions (pH, H_2_O_2_ concentration, and RhB concentration) on the removal of RhB by the photo-Fenton-like system (**a**) [H_2_O_2_] 90 mM and [RhB] 40 mg/L, (**b**) pH 2.5 and [RhB] 40 mg/L, (**c**) [H_2_O_2_] 90 mM and pH 2.5 at time 80 min and T. 303 K.

RhB was subjected to photo-Fenton catalytic degradation in a batch reactor with different parameters, such as initial RhB concentration, medium pH, and oxidant loading, to maximize the process’s efficiency. The findings are presented in Fig. 8. The amount of accessible active sites on the catalyst is impacted by changes in the medium pH. Additionally, these changes might affect the charge of the pollutants and, as a result, the rate of removal at the catalysts’ active sites^58^. The impact of pH on the rate of photo-Fenton degradation in an acidic pH range (2–6) at room temperature, assuming a moderate fixed dye concentration of 40 mg/L, is depicted in Fig. 8a. It is evident that as the pH rises to 6, the effectiveness of RhB degradation diminishes. Research has demonstrated that the pH of the solution affects the production of HO^•^. The HO^•^ created by the Fenton reagent is predicted to demonstrate the most oxidative power at pH 3. It is important to note from Fig. 8a that the pH range shown to work best for Fenton’s reaction is about 2.5. However, the reaction is less effective at neutral or nearly neutral pH values. The substantial impact of reduced pH on metal oxidation may explain this. The relationship between Fe^2+^ oxidation and [HO^−^]^2^ in aqueous environments is widely established^59^. Consequently, the oxidation of Fe^2+^ and Cu^+^ at neutral or nearly neutral pH (e.g., pH 4) and the ensuing production and precipitation of insoluble metal hydroxides are essential processes. Because of this, there is very little Fe^2+^ available, and iron in this form breaks down H_2_O_2_ into oxygen and water^60^, consequently, the oxidation rate decreases. Additionally, during the photo-Fenton process, the precipitated hydroxide reduces the radiation’s transmission^59^. As the pH drops, ^•^OH’s oxidation potential rises, strengthening its oxidation capacity^61^.

Figure 8b illustrates the impact of different concentrated hydrogen peroxide dosages on the photo-Fenton degradation of RhB dye. Compared to other hydrogen peroxide doses, the elimination capacity of the 90 mM hydrogen peroxide dosage was faster. A significant amount of hydrogen peroxide may guarantee a sufficient amount of HO^•^ to break down RhB. When the initial H_2_O_2_ dosage was 90 mM, the removal efficiencies were 41.5%, 67.2%, and 94.2% for BAT, CuFe_2_O_4_, and BAT/CMC/CuFe_2_O_4_, respectively. However, the efficacy of RhB breakdown begins to decline at hydrogen peroxide dosages greater than 90 mM. This is because excessive H_2_O_2_ scavenges HO^•^ radicals in solution, impeding the oxidative cycle’s propagation stage (H_2_O_2_ + HO^•^ $\to$H_2_O + HO_2_^•^).

Given that it is a crucial variable in the degradation processes of organic pollutants, the impact of the initial concentration of RhB dye, ranging from 20 to 100 mg/L, on the degradation efficiency was examined. The findings are displayed in Fig. 8c. As RhB concentrations increased, the effectiveness of its degradation tended to diminish. One reason for this behavior might be that as dye concentration increases, more dye molecules collide with each other, while fewer dye molecules collide with HO• radicals^62^. The reaction rate is thus slowed down. Retardation in the degradation process is observed because the solution’s visible light transmittance decreases with concentration, making it more inaccessible to UV/Vis. Radiation during photo-Fenton processes. This means that lower photons accomplish the catalyst surface and activate it to generate HO^•^ and O_2_^•^ radicals^63^.

**Fig. 9:**
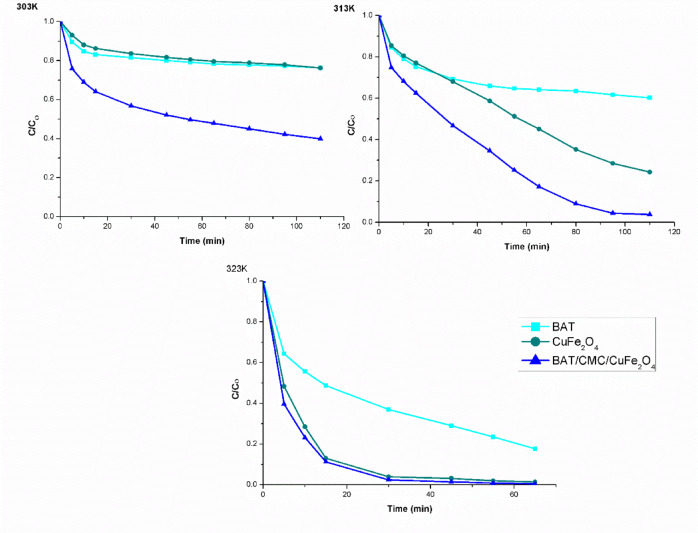
Effect of time on RhB dye degradation ratios through different Fenton systems (operating conditions: [H_2_O_2_] 90 mM, [RhB] 40 mg/L, and pH ~ 2.5).

**Fig. 10:**
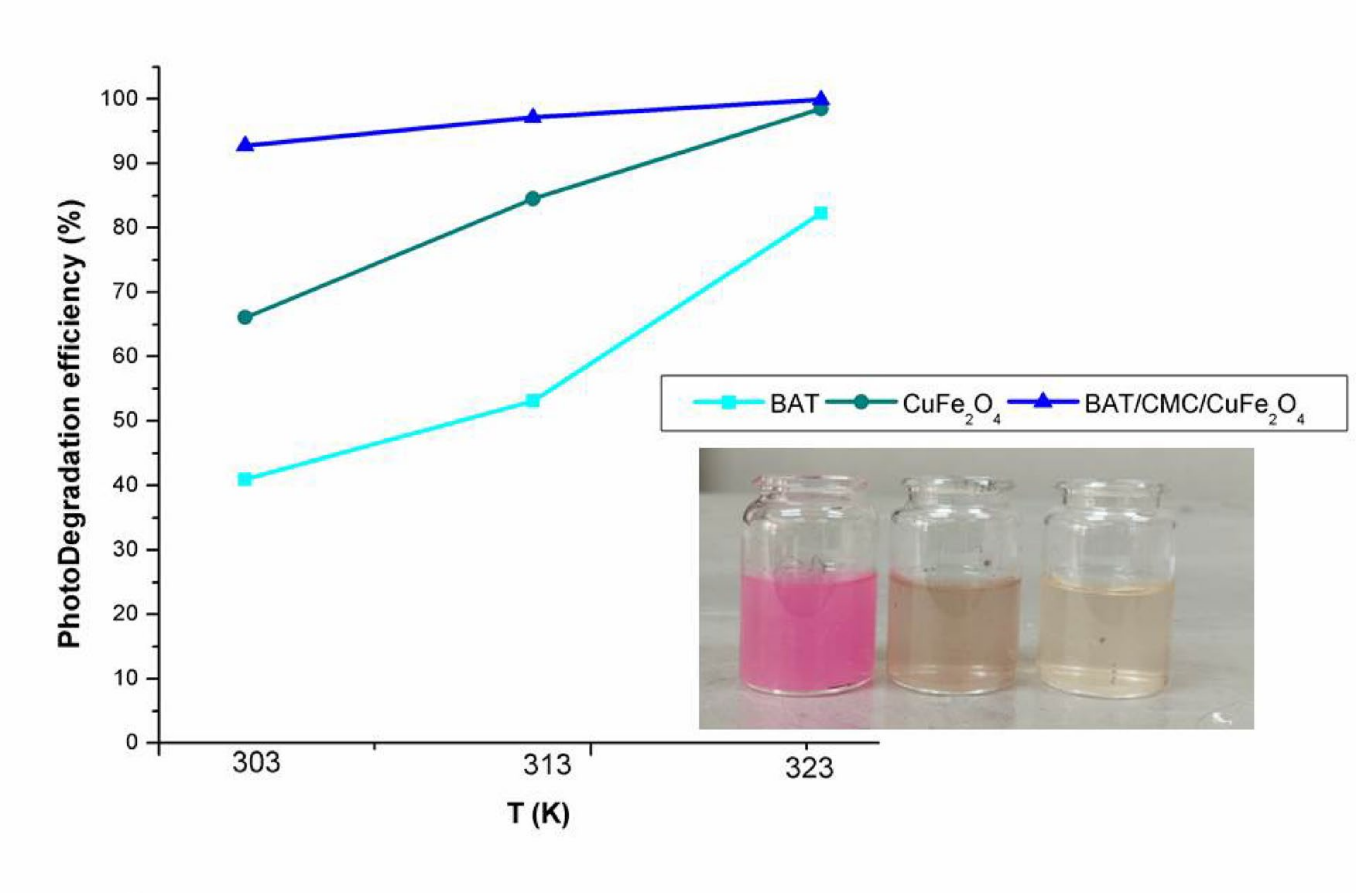
Effect of temperature on degradation efficiency of RhB dye degradation with a photograph of maximum degradation efficiency by the prepared catalysts at 323 K (operating conditions: [H_2_O_2_] 90 mM, [RhB] 40 mg/L, and pH 2.5, and time 80 min).

The two most significant variables influencing photo-Fenton degradation are the temperature and contact duration between RhB and the catalysts. The rate of RhB degradation by the photo-Fenton reaction for BAT, CuFe_2_O_4_, and BAT/CMC/CuFe_2_O_4_ is demonstrated in Fig. 9 concerning applied temperature changes (303, 313, and 323 K) using 50 mL of dye solution with an initial concentration of 40 mg/L, 0.05 g catalyst dosage, pH ~ 2.5, and 90 mM of [H_2_O_2_] at various time intervals. RhB photo-Fenton degradation increases gradually with longer irradiation times, as shown in Fig. 9, and eventually stabilizes at a certain point^62^. The maximum degradation rate is observed after 80 min at 303 K when RhB dye is degraded using a BAT/CMC/CuFe_2_O_4_ catalyst. One important operational parameter in Fenton processes is reaction temperature^64^. Thus, to determine the apparent activation energy, the impact of reaction temperature on RhB decolorization was assessed. In the three systems under consideration, increasing the temperature promoted decolorization in the following order: BAT/CMC/CuFe_2_O_4_ > CuFe_2_O_4_ > BAT. The degradation proceeded smoothly and increased until it attained stability at 303 K. Conversely, once the temperature rose, the rate of degradation accelerated significantly until it achieved its maximum between 313 and 323 K, as displayed in Fig. 9. The reason for this increase is that the catalyst and hydrogen peroxide reacted more quickly at higher temperatures, leading to the production of more reactive oxygen species, including the HO^•^ radical. Such reactions appear to be endothermic based on these findings. Decolorization occurred more rapidly at higher temperatures than at lower ones. After 60 min of reaction at 323 K, RhB was almost completely decolorized in both the CuF_2_O_4_ and BAT/CMC/CuF_2_O_4_ systems as obvious in Fig. 10.

### Photo-Fenton catalytic reaction mechanism

The subsequent equation identifies an essential stage in the conventional Fenton-like reaction as it generates the powerful oxidizing HO• radical:

9 $${\varvec{F}\varvec{e}}^{2+}+{\varvec{H}}_{2}{\varvec{O}}_{2}\to{\varvec{F}\varvec{e}}^{3+}+{\varvec{H}\varvec{O}}^{-}+{\varvec{H}\varvec{O}}^{\varvec{\cdot}}$$

Under dark conditions and in the absence of alternative Fe^3+^-reducing species, regeneration of Fe^2+^ is the rate-determining step^65^.

10 $${\varvec{F}\varvec{e}}^{3+}+{\varvec{H}}_{2}{\varvec{O}}_{2}\to{\varvec{F}\varvec{e}}^{2+}+{\varvec{H}\varvec{O}}_{2}^{\varvec{\cdot}}+{\varvec{H}}^{+}$$

11 $${\varvec{F}\varvec{e}}^{2+}+{\varvec{H}\varvec{O}}^{\varvec{\cdot}}\to{\varvec{F}\varvec{e}}^{3+}+{\varvec{H}\varvec{O}}^{-}$$

Fenton-type reactions can be induced by transition metal ions other than Fe(II). In this context, copper may react with H_2_O_2_ in both of its oxidation states to create HO_2_^•^ and HO^•^ radicals^66^.

12 $${\varvec{C}\varvec{u}}^{2+}+{\varvec{H}}_{2}{\varvec{O}}_{2}\to{\varvec{C}\varvec{u}}^{+}+{\varvec{H}\varvec{O}}_{2}^{\varvec{\cdot}}+{\varvec{H}}^{+}$$

13 $${\varvec{C}\varvec{u}}^{+}+{\varvec{H}}_{2}{\varvec{O}}_{2}\to{\varvec{C}\varvec{u}}^{2+}+{\varvec{H}\varvec{O}}^{-}+{\varvec{H}\varvec{O}}^{\varvec{\cdot}}$$

According to prior studies^13,67^, electron transfer across the interface can be accelerated by the collaboration between the redox pairs of iron (Fe^3+^/Fe^2+^) and copper (Cu^+^/Cu^2+^), which assists in the fast reduction of Fe^3+^. As reported by Sun et al.^14^, the Fe^2+^ species of the Fe–Cu bimetallic catalyst is predominantly regenerated by the interaction of Fe^3+^ with Cu^+^ (Eq. 14) instead of Fe^3+^ being reduced by H_2_O_2_ (Eq. 10).

14 $${\varvec{C}\varvec{u}}^{+}+{\varvec{F}\varvec{e}}^{3+}\to{\varvec{C}\varvec{u}}^{2+}+{\varvec{F}\varvec{e}}^{2+}$$

When exposed to ultraviolet (UV) or visible light, the processes of (H_2_O_2_/Fe^2+^) and (H_2_O_2_/Cu^2+^) in Photo-Fenton-like reactions can be considerably accelerated. The ultraviolet portion of the electromagnetic spectrum is between 100 and 400 nm, whereas the visible part extends from around 400 nm to 760 nm^15^. Since Fe^2+^ ions are regenerated from Fe^3+^ by photo-reduction, photo-irradiation inhibits the agglomeration of Fe^3+^ ions in the system^68,69^. It was discovered that subjecting Fenton reaction systems to UV/Visible light substantially sped up the rate at which a range of contaminants degraded. The main cause of this behavior under irradiation is the photochemical reduction of Cu(II) to Cu(0) and Fe(III) back to Fe(II). The net reactions may be expressed as follows:

15 $${\varvec{C}\varvec{u}\varvec{F}\varvec{e}}_{2}{\varvec{O}}_{4}+\varvec{h}\varvec{\upsilon}\to{\varvec{e}}^{-}+{\varvec{h}}^{+}$$

16 $${\varvec{H}}_{2}{\varvec{O}}_{2}+{\varvec{e}}^{-}\to{\varvec{H}\varvec{O}}^{\varvec{\cdot}}+{\varvec{H}\varvec{O}}^{-}$$

17 $${\varvec{F}\varvec{e}}^{3+}+{\varvec{e}}^{-}\to{\varvec{F}\varvec{e}}^{2+}$$

18 $${\varvec{C}\varvec{u}}^{2+}+{\varvec{e}}^{-}\to{\varvec{C}\varvec{u}}^{+}$$

19 $${\varvec{C}\varvec{u}}^{+}+{\varvec{e}}^{-}\to{\varvec{C}\varvec{u}}^{0}$$

20 $${\varvec{h}}^{+}+{\varvec{H}}_{2}{\varvec{O}}_{2}\to{\varvec{H}\varvec{O}}_{2}^{\varvec{\cdot}}+{\varvec{H}}^{+}$$

21 $${\varvec{h}}^{+}+{\varvec{F}\varvec{e}}^{2+}\to{\varvec{F}\varvec{e}}^{3+}$$

22 $${\varvec{h}}^{+}+{\varvec{C}\varvec{u}}^{+}\to{\varvec{C}\varvec{u}}^{2+}$$

23 $${\varvec{h}}^{+}+\varvec{o}\varvec{r}\varvec{g}\varvec{a}\varvec{n}\varvec{i}\varvec{c}\varvec{c}\varvec{o}\varvec{m}\varvec{p}\varvec{u}\varvec{n}\varvec{d}\varvec{s}\to\varvec{o}\varvec{x}\varvec{i}\varvec{d}\varvec{i}\varvec{z}\varvec{e}\varvec{d}\varvec{p}\varvec{r}\varvec{o}\varvec{d}\varvec{u}\varvec{c}\varvec{t}\varvec{s}$$

**Fig. 11:**
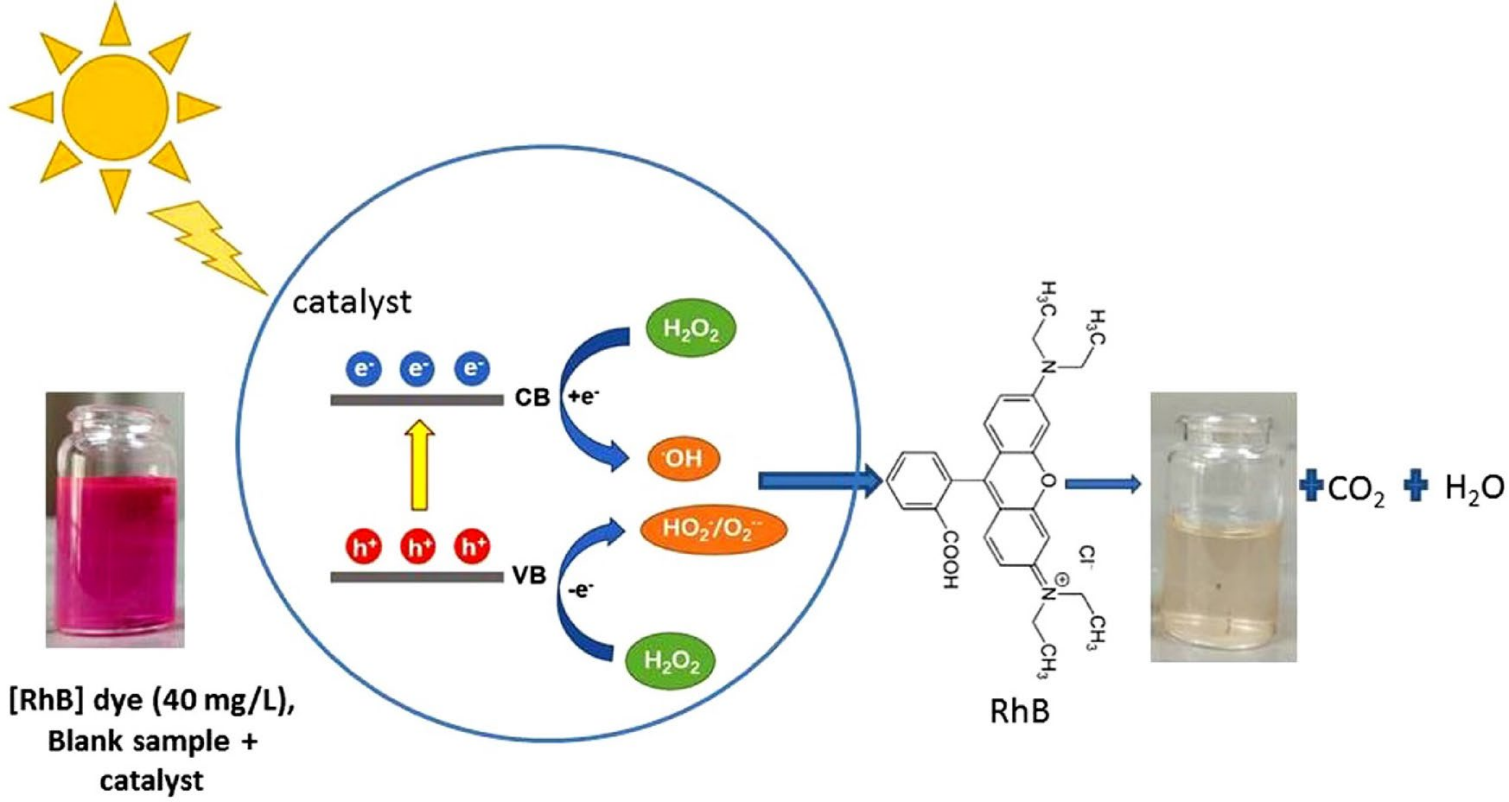
The common mechanism of photo-Fenton catalytic degradation of RhB dye.

In the metal-free photo-Fenton-like catalyst (BAT), a significant quantity of H_2_O_2_ is absorbed by the carbonyl and amino groups through strong hydrogen bonds (O–H....N, O–H.....O) at the electron-rich centers, where it is quickly reduced to HO•^70^. Moreover, the photo-generated electron and hole pairs (e^−^/h^+^) split off, breaking down organic pollutants or further reducing the H_2_O_2_ molecules that have been adsorbed to create oxidant species^71,72^. Through these processes, contaminants rapidly undergo mineralization and degradation across a broad pH range, leading to better H_2_O_2_ utilization efficiency. Figure 11 can be used to state the dye degradation mechanism by photo-Fenton-like catalysts.

Table 1 records that the photo-Fenton catalytic activity of the prepared catalysts, BAT, CuFe_2_O_4_, and BAT/CMC/CuFe_2_O_4_, demonstrated superior Fenton catalytic activity compared to conventional Fenton degradation in a dark environment. This was achieved by employing the optimized H_2_O_2_ dosage, pH, and RhB dye concentration at 303 K for 80 min. Photo-Fenton tests showed that pure BAT removed only 41.5% of the RhB dye, CuFe_2_O_4_ degraded up to 67.2% of the RhB dye, while their composite with CMC, BAT/CMC/CuFe_2_O_4_, exhibited the best degradation performance under solar irradiation, degrading up to 94.2% of the dye. This is because CMC serves as a large support, accommodating and immobilizing the BAT and CuFe_2_O_4_ particles for easy catalyst recycling and accelerating the transport of induced electrons for better charge separation. As a result, redox cycles and catalytic activities are accelerated and increased^15^. The degradation decreased particularly while performing the conventional Fenton-degradation approach. This is demonstrated by the fact that HO^•^ radicals and lower-state metal cations (Fe^2+^/Cu^+^) may continue to regenerate when exposed to solar activation. Due to these considerations, the photo-Fenton technique for RhB dye degradation proceeds more quickly than the conventional Fenton procedure.

**Table 1:** A comparative study between conventional- and photo-Fenton catalytic degradation for RhB dye ([H_2_O_2_] 90 mM, pH 2.5, [RhB] 40 mg/L, T 303 K, and time 80 min).

	Degradation efficiency (%)		
	BAT	CuFe_2_O_4_	BAT/CMC/CuFe_2_O_4_
Conventional-Fenton like reaction	15.1	18	39.5
Photo-Fenton like reaction	41.5	67.2	94.2

### Kinetic modeling

Exposing a catalyst to ultraviolet (UV) or visible light significantly speeds up Photo-Fenton-like reactions, particularly those involving (H_2_O_2_/Fe^2+^) and (H_2_O_2_/Cu^2+^). Fenton reactions typically oxidize organic molecules in two stages. The rapid stage occurrs due to the direct interaction between Fe^2+^, Cu^2+^ and H_2_O_2_. The subsequent, slower stage happens because the resultant Fe^3+^ and Cu^+^ accumulate, and the generation of Fe^2+^ and Cu^2+^ species by UV or H_2_O_2_ is limited.

**Fig. 12:**
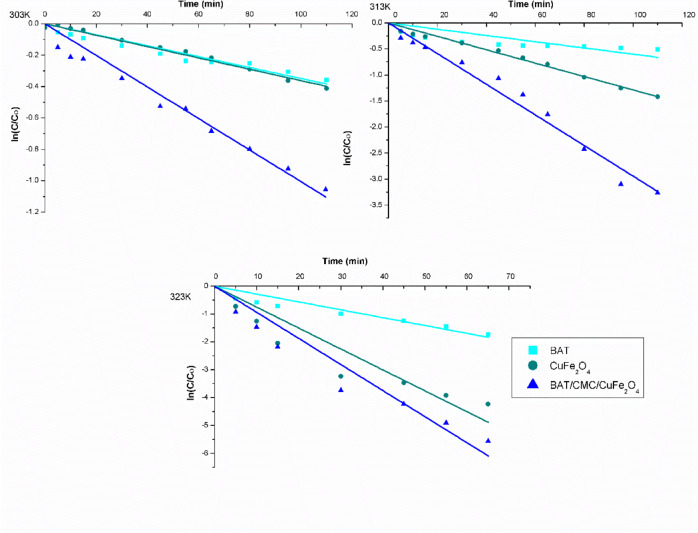
1st order kinetic study for oxidative degradation of RhB dye by prepared catalysts at different temperatures ([H_2_O_2_] 90 mM, [RhB] 40 mg/L, and pH 2.5).

Plotting $\text{ln}\left(\frac{\text{C}}{{\text{C}}_{^\circ}}\right)$Versus time, as presented in Fig. 12, revealed a straight line with a negative slope. The slope of this line reflects the apparent value of the first-order rate constant (K_app_, min^− 1^) for the organic target compound decomposition. Table 2 presents an outline of the identified kinetic parameters. From Table 2, estimates of correlation coefficients, R^2 ^for pseudo-first-order are higher than those for pseudo-second-order and closer to 1. Across the applied temperatures, the apparent first-order rate constant (K_app_) for prepared samples in that order; BAT/CMC/CuFe_2_O_4_ > CuFe_2_O_4_ > BAT is ascribed to their high activity. The values of K_app_ rate constant increase with temperature from 0.004 to 0.03 for BAT, from 0.0036 to 0.075 for CuFe_2_O_4_, and from 0.01 to 0.09 for BAT/CMC/CuFe_2_O_4_, attributed to the increase in reaction rate with the increase in temperature^73^.

### Thermodynamic modeling

**Fig. 13:**
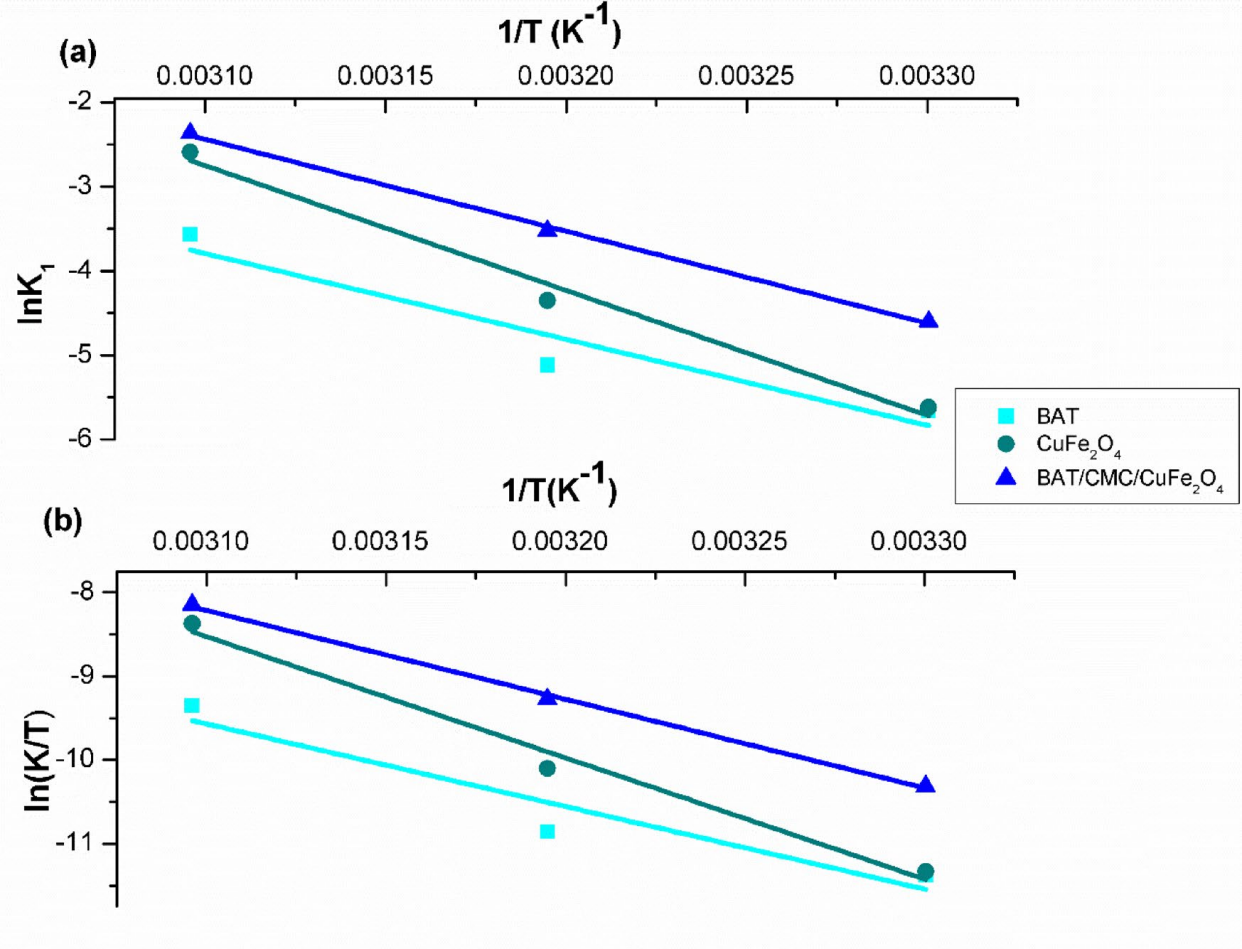
(**a**) Arrhenius and (**b**) Eyring-Polanyi plots of photo-Fenton catalytic degradation of RhB dye by prepared catalysts ([H_2_O_2_] 90 mM, [RhB] 40 mg/L, pH 2.5, and time 80 min).

Upon evaluating the data, Table 2 demonstrates the effective use of the Arrhenius and Eyring-Polanyi models based on the higher R^2^ values (> 0.97) for the two prepared samples BAT/CMC/CuFe_2_O_4_ and CuFe_2_O_4_. Besides, the computed values of Ea (84.7, 123.1, 90.7 kJ/mol) in the photo-Fenton degradation of RhB dye catalyzed by BAT, CuFe_2_O_4_, and BAT/CMC/CuFe_2_O_4_ are more than 20 kJ/mol, indicating the chemical nature of the degradation process^74^. Additionally, the values of ΔH* are positive, pointing out that photo-Fenton degradation of RhB dye is endothermic^75^. The ΔS* values were calculated to be -0.023 for BAT, 0.105 for CuFe_2_O_4_, and 0.0074 for BAT/CMC/CuFe_2_O_4_. The reduction in the randomness of RhB molecules at the solid surface of BAT, implying an irreversible tendency for the process, contributes to the negative values of ΔS*. In contrast, the increase in randomness of RhB molecules at the surface of the other two prepared catalysts accounts for the positive values. Moreover, the values of ΔG* are positive, evidencing that the RhB degradation process is non-spontaneous (Fig. 13)^76^.

**Table 2:** Kinetic and thermodynamic parameters of RhB dye catalytic degradation using prepared catalysts.

Catalyst		BAT			CuFe_2_O_4_			BAT/CMC/CuFe_2_O_4_		
		303 K	313 K	323 K	303 K	313 K	323 K	303 K	313 K	323 K
Kinetic Parameters										
Pseudo 1st order	K_1_(min^− 1^)	0.004	0.006	0.03	0.0036	0.013	0.075	0.01	0.03	0.09
	R^2^	0.976	0.985	0.969	0.997	0.995	0.962	0.989	0.99	0.972
Pseudo 2nd order	K_2_(min^− 1^)	0.007	0.02	0.2	0.008	0.13	3.3	0.04	0.7	0.8
	R^2^	0.712	0.795	0.957	0.814	0.777	0.951	0.931	0.809	0.922
Thermodynamic Parameters		84.7			123.1			90.74		
E_a_ (k.J/mol)										
R^2^		0.9191			0.9747			0.9968		
ΔH* (k.J/mol)		82.1			120.5			88.14		
ΔS* (k.J/mol.K)		− 0.023			0.105			0.0074		
R^2^		0.915			0.9738			09967		
ΔG* (k.J/mol)		89	89.3	89.5	88.7	87.6	86.6	85.9	85.8	85.7

### N_2_-Adsorption isotherms, BET-Surface area, and pore size distribution

**Fig. 14:**
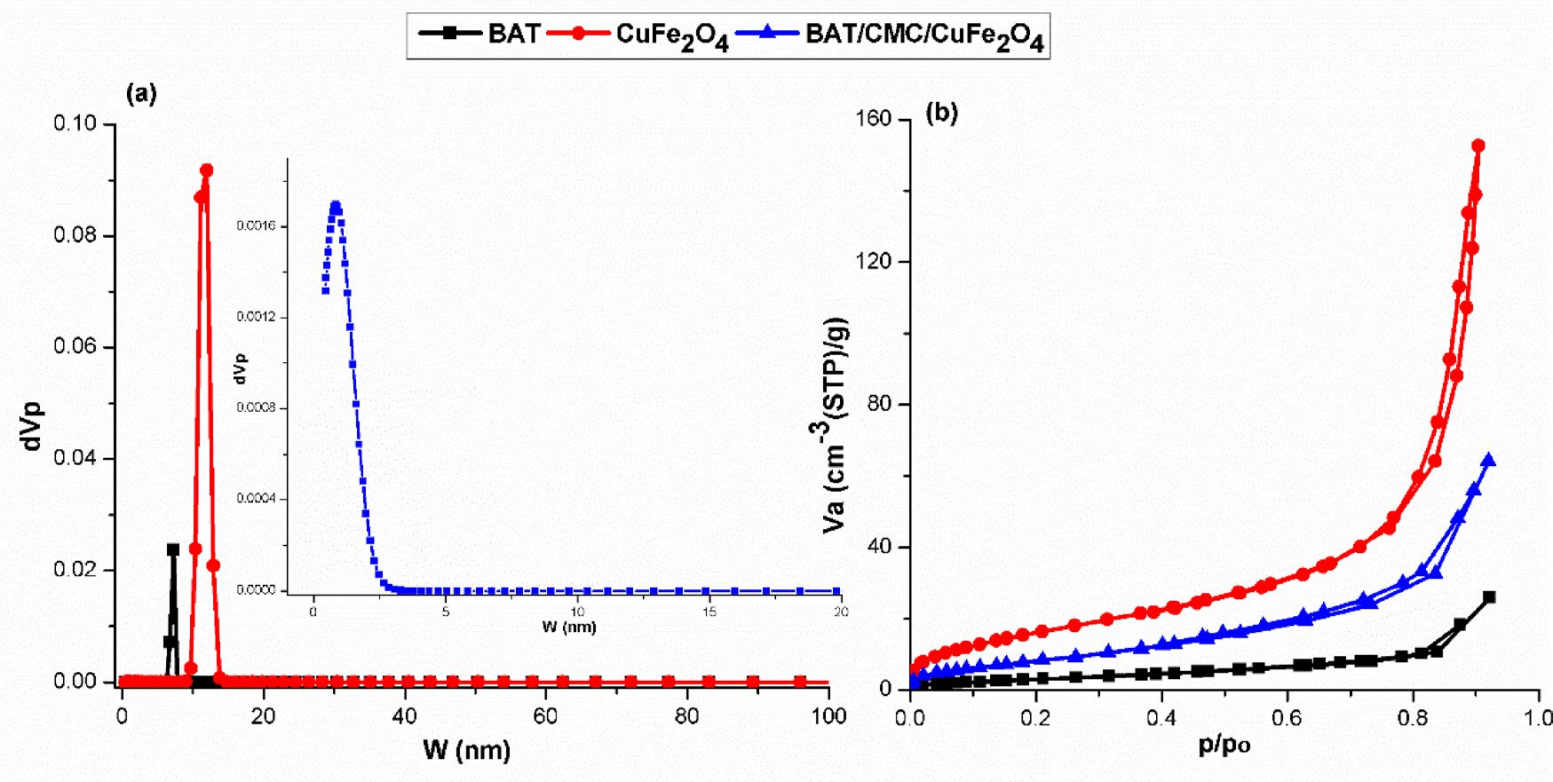
(**a**) Pore size distribution curves, and (**b**) N_2_ adsorption-desorption isotherms of the as-prepared catalysts.

The Brunauer-Emmett-Teller (BET) equation and non-local density functional theory (NLDFT) were applied to figure out the specific surface area (S_BET_) and pore size distribution (PSD) curves, respectively, to explore the effects of produced CuFe_2_O_4_ on the surface characteristics of assessed catalysts. Furthermore, Fig. 14 clarifies N_2_ adsorption − desorption isotherms. The pore size distribution (Fig. 14a) assumed that the mesopores for BAT and CuFe_2_O_4_ comprised a cylindrical slit-shaped pores, with a pore size distribution peak around 7–12 nm. The pore size distribution peak for the BAT/CMC/CuFe_2_O_4_ composite was around 0.5–1.5 nm when CMC and CuFe_2_O_4_ were incorporated confirming the microporous structure. According to the International Union of Pure and Applied Chemistry (IUPAC) classification, the evaluated catalysts have a Type IV isotherm and a Type H1 hysteresis loop, demonstrating the presence of mainly micro/mesoporous structure and slit-shaped pores^77,78^, as exhibited by the N_2_ adsorption − desorption isotherms (Fig. 14b).

The surface and pore properties of evaluated catalysts are displayed in Table 3. It can be observed that the S_BET_ of CuFe_2_O_4_ showed the highest value of 64 m^2^/g. As the CuFe_2_O_4_ content was inserted into the composite catalyst, the S_BET_ of amide linkage polymer-based catalysts increased from 12.7 m^2^/g for BAT into 35.6 m^2^/g for BAT/CMC/CuFe_2_O_4_. The increase of S_BET_ may be due to the insertion of CuFe_2_O_4_ with its high surface area; however, the decrease of S_BET_ compared to CuFe_2_O_4_ may be attributed to the aggregation of CMC inside composite structure^79^.

**Table 3:** Surface textural properties and isoelectric pH of prepared catalysts.

Textural Characteristics		BAT	CuFe_2_O_4_	BAT/CMC/CuFe_2_O_4_
S_BET_ (m^2^/g)		12.7	64	35.6
Mean pore diameter (nm)		12.5	14.8	11
Total pore volume (cm^3^/g)		0.04	0.24	0.1
Isoelectric pH (pH_pzc_)		3.5	6.6	6.7

### Reusability

**Fig. 15:**
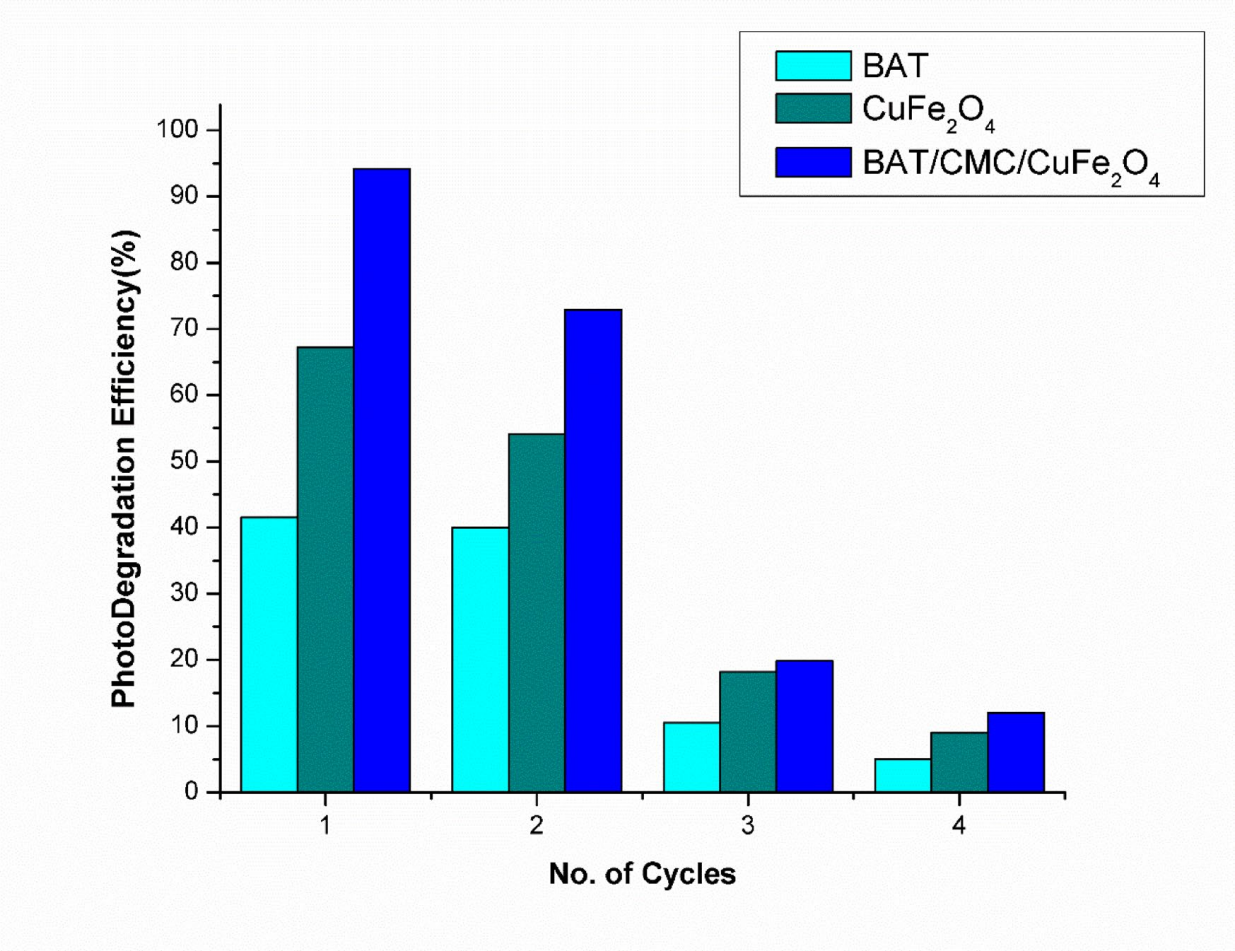
Reusability cycles of prepared catalysts for the photo-Fenton catalytic degradation of RhB dye ([RhB] 40 mg/L, [H_2_O_2_] 90 mM, pH 2.5, T. 303 K, and time 80 min).

As demonstrated in Fig. 15, the stability and recycling studies for the prepared catalysts towards the photo-Fenton process’s degradation of RhB dye were evaluated over four consecutive cycles. Following the photo-Fenton catalytic reaction, double-distilled water was used to filter and wash the produced samples: BAT, CuFe_2_O_4_, and BAT/CMC/CuFe_2_O_4_. Under optimal conditions, evaluations were carried out four times. As apparent in Fig. 15, following the fourth test, the RhB photo-Fenton catalytic degradation efficiency for BAT, CuFe_2_O_4_, and BAT/CMC/CuFe_2_O_4_ is around 5%, 9%, and 12%, respectively. This finding claimed that the instability of prepared catalysts for further reproduction and the loss of certain active sites and porosity may have contributed to the decrease in their catalytic activity^74^. Additionally, environmental impact can hinder the regeneration of Fe^2+^ and Cu^+^, required for stability during the degradation mechanism. Changes in surrounding conditions may cause instability and decrease removal efficiency. The instability challenges are affected by increasing temperature, changes in pH, high concentrations of contaminants, and equilibrium contact time. It may require a continuous regeneration process to maintain efficiency over a longer period while keeping the surrounding conditions constant.

## Computational insights of prepared catalysts

**Fig. 16:**
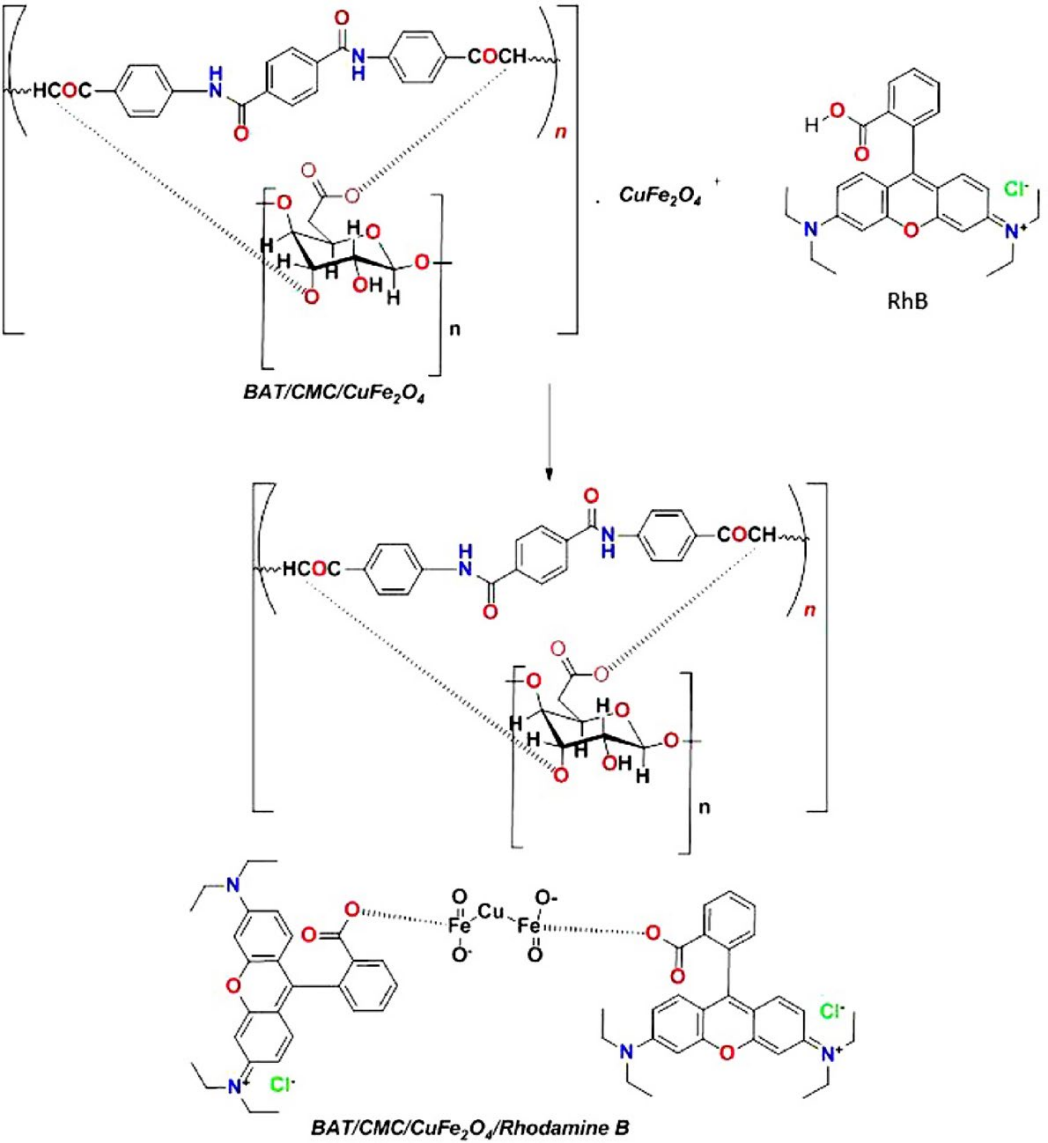
Proposed chemical interaction between the BAT/CMC/CuFe_2_O_4_ and RhB dye.

In this study, we optimized the BAT/CMC/CuFe_2_O_4_, RhB dye, and BAT/CMC/CuFe_2_O_4_/RhB utilizing Gaussian (09)^80,81^ through DFT/B3LYP/LAN2DZ(G) basis set. Moreover, the physical characteristics used in the optimization of molecular structures of BAT/CMC/CuFe_2_O_4_, RhB, and BAT/CMC/CuFe_2_O_4_/RhB concerned (σ) absolute softness^82^, (χ) electro-negativities^83^, (*Δ N*_*max*_) electronic charge^84^, (*η*) absolute hardness^85^, (*ω*) global electro-philicity^86^, (*S*) global softness^87^, and (*Pi*) chemical potential^88^ from Eqs. (24–31)^49,89–91^ which are declared in Table 4 and Fig. 16.

In this proposed mechanism, the polar carbonyl (C = O) group in the polymer’s amide structure forms hydrogen bonds with the amine group of RhB dye, facilitating the adsorption energy of the dye. Additionally, the polymer and RhB’s aromatic rings interact through π–π stacking, which further improves removal efficiency. When RhB is present in its ionic form or the polymer contains charged groups, electrostatic attractions also play a role. These interactions promote the efficient trapping of RhB from aqueous solutions. Furthermore, two RhB dye molecules interact with the bimetallic oxide. CuFe_2_O_4_ readily chelates when -COOH is present, engaging in hydrogen bonding interactions. This indicates a chemical reaction, confirming the oxidation of Cu and Fe metal ions and explaining the absence of the RhB color.

**Table 4:** Equations of physical descriptors.

${\Delta}\text{E}={\text{E}}_{\text{L}\text{U}\text{M}\text{O}}-{\text{E}}_{\text{H}\text{O}\text{M}\text{O}}$	(24)	${\upchi}=\frac{{-(\text{E}}_{\text{H}\text{O}\text{M}\text{O}}+{\text{E}}_{\text{L}\text{U}\text{M}\text{O}})}{2}$	(25)
${\upeta}=\frac{\left({\text{E}}_{\text{L}\text{U}\text{M}\text{O}}-{\text{E}}_{\text{H}\text{O}\text{M}\text{O}}\right)}{2}$	(26)	${\upsigma}=\frac{1}{{\upeta}}$	(27)
Pi = − Ӽ	(28)	$\text{S}=\frac{1}{2}{\upeta}$	(29)
${\upomega}=\frac{\text{P}{\text{i}}^{2}}{2}$	(30)	${\Delta}\text{N}\text{m}\text{a}\text{x}=-\frac{\text{P}\text{i}}{{\upeta}}$	(31)

**Fig. 17:**
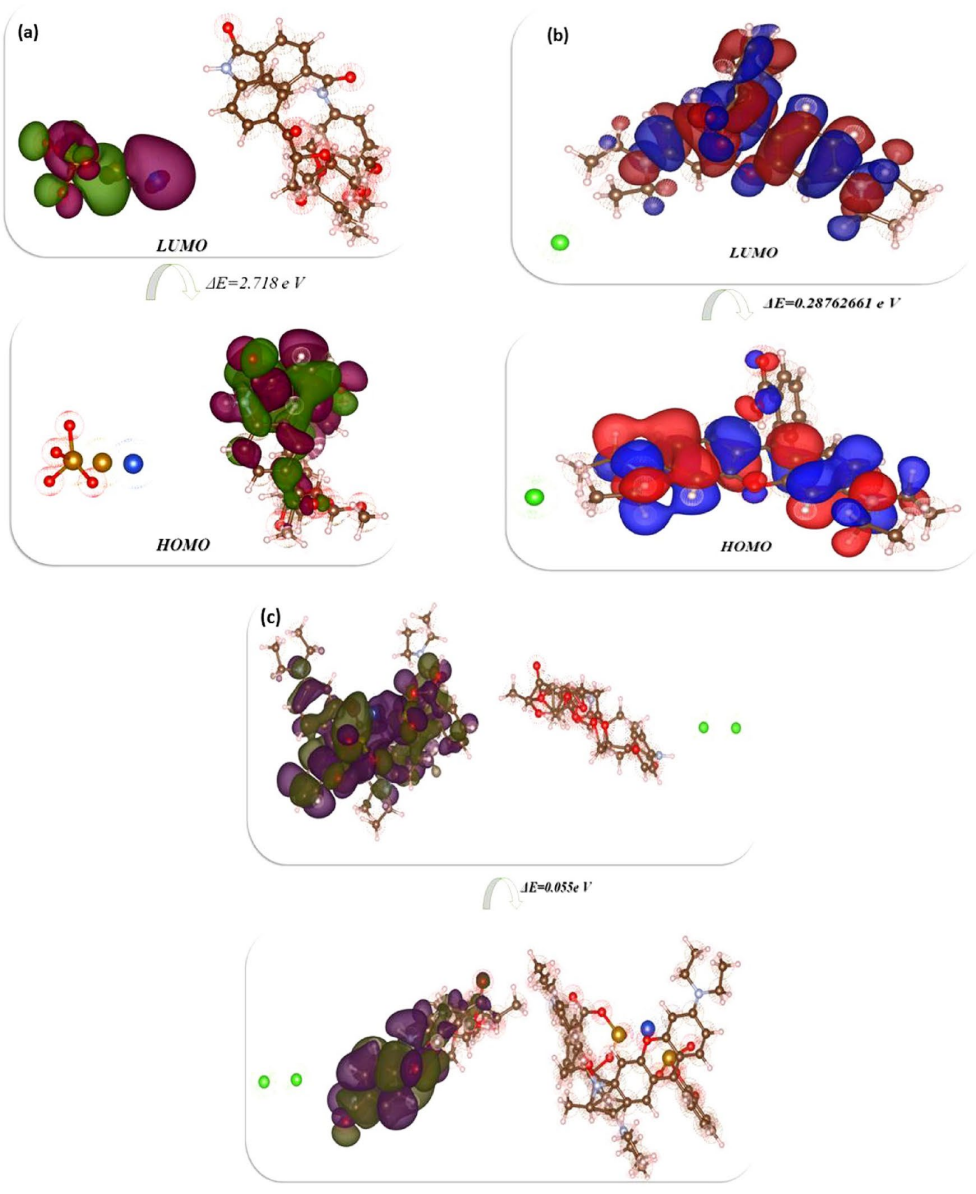
HOMO and LUMO energy configuration of; (**a**) BAT/CMC/CuFe_2_O_4_, (**b**) RhB, and (**c**) BAT/CMC/CuFe_2_O_4_/RhB: Visualized with Gauss view 5 (version number 5, and URL link. https://media.dynauie6.sbs/gauss+view+5+linux.torrent.zip), Gaussian 09 (version number 9, and URL link. https://gaussian.com/glossary/g09/**)**, and VESTA (version number 3, and URL link. https://jp-minerals.org/vesta/en/) softwares.

Studies using Density Functional Theory (DFT) provide important molecular-level insights into these interactions, enabling the prediction of electronic behavior, binding energy, and adsorption stability. Ultimately, current computational studies enhance environmental remediation efforts by expanding our knowledge of basic principles and guiding the development of more effective and customized amide polymer adsorbents for dye removal. The synthesized compounds were optimized through DFT investigation to determine their physical properties and their interaction with RhB employing the DFT/B3LYP/LAN2DZ(G) basis set, as displayed in Fig. 17 and Table 5. Firstly, the optimized structure of BAT/CMC/CuFe_2_O_4_ showed a total energy of -72242.892853 e V (-1665960.2609 kcal/mol), indicating the stability of BAT within the CMC pocket in the presence of metal oxide, which enhanced its activity. Electron delocalization was observed in HOMO in the BAT/CMC, while in LUMO it was observed in CuFe_2_O_4_, with a difference between them of 2.718 e V. Its dipole moment showed a high value of 11.7683 Debye due to the presence of CuFe_2_O_4_ with positive charges that can easily separate^92^, as shown in Fig. 17a. Furthermore, the electronegativity of BAT/CMC/CuFe_2_O_4_ is 8.512 e V due to the dual charges of iron with oxide and the four negative charges of oxygen. Additionally, its hardness is 1.359 e V due to strong bonding with the CMC, and its chemical potential (Pi) is -8.512 e V, resulting from the metal oxide on the polymer.

The optimized structure of RhB also showed a non-planar configuration and the presence of positive and negative charges on nitrogen and chlorine. Its total energy is -50893.387031 e V (-1173629.0864 kcal/mol), with localization of chargers on the nitrogen and the xanthene, but not on the chlorine atom, which has negative charges. The band gap energy was 0.288 e V, as demonstrated in Fig. 17b. Its dipole moment was 31.607 Debye, with Cl^−^ easily charged and its electro-negativities (χ) at 3.765 e V, related to N^+^, and Cl^−^, which neglected each other. The absolute hardness (η) of RhB showed a low value of 0.144 e V, while its softness exhibited a high value of 6.953 e V, indicating smooth interaction. Finally, the reactivity of RhB with BAT/CMC/CuFe_2_O_4_ showed a total energy of -105480.7705 e V (-2432443.732 kcl/mol) and delocalization in the HOMO with BAT/CMC, while in the LUMO with RhB/BAT/CMC/CuFe_2_O_4_ the band energy gap between HOMO-LUMO was 0.027 e V, indicating a small band gap energy that facilitates electron acceleration between them, as displayed in Fig. 17c. It is more active, confirmed by the activity with RhB and the disappearance of its color. Its dipole moment was 54.9876 Debye, a high value due to the presence of more separated charges on atoms. The electronegativity was 4.908 e V, representing the attachment between them and the presence of free Cl^−^ atoms, as well as increased surface softness in the presence of RhB with 36.570 e V^93^.

**Table 5:** The physical descriptors for optimum prepared catalyst, BAT/CMC/CuFe_2_O_4_, RhB dye, and BAT/CMC/CuFe_2_O_4_/RhB utilizing the DFT/B3LYP/LAN2DZ(G) basis set.

Physical Descriptors	BAT/CMC/CuFe_2_O_4_	RhB	BAT/CMC/CuFe_2_O_4_ /RhB
E_T_ (au)	− 2654.876	− 1870.297	− 3876.345
E_HOMO_ (e V)	− 9.871	− 3.908	− 4.9352
E_LUMO_ (e V)	− 7.153	− 3.620	− 4.88051
E_g_ (e V)	2.718	0.288	0.055
µ (D)	11.768	31.607	54.987
χ (e V)	8.512	3.765	4.908
η (e V)	1.359	0.144	0.027
σ (e V)	0.736	6.953	36.570
Pi (e V)	− 8.512	− 3.765	− 4.908
S (e V)	0.368	3.477	18.285
ω (e V)	26.663	49.272	440.429
ΔN_max_	2.264	26.176	179.479

**Fig. 18:**
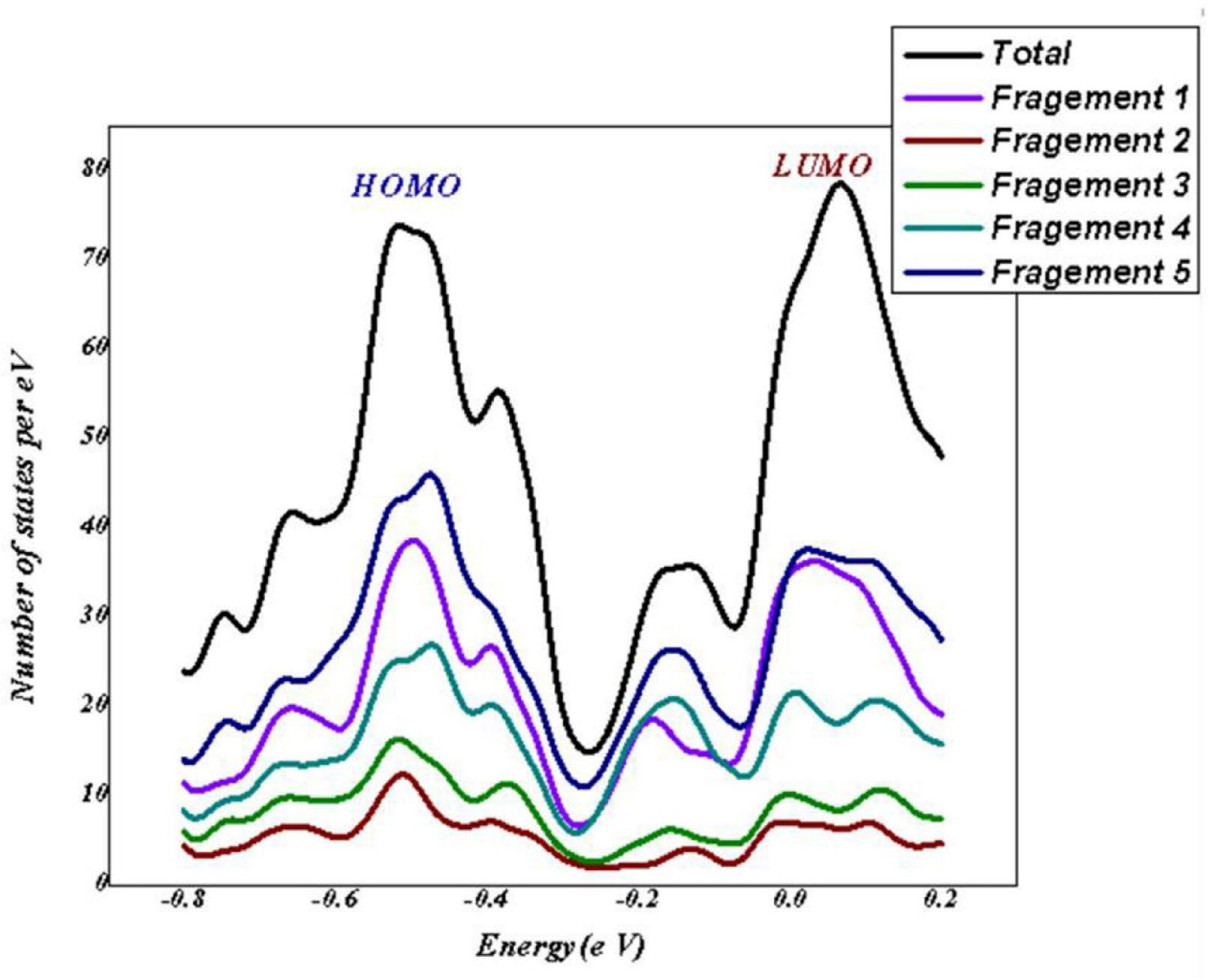
The density of states plots including total and fragemnts of BAT/CMC/CuFe_2_O_4_/RhB: Visualized with Multiwifn software (version number 3.7, and URL link. http://sobereva.com/multiwfn/).

**Total Density of States:** A key idea in computational chemistry is the density of states (DOS), which offers crucial information about how the electron energy levels are distributed within a molecule or substance. This fundamental concept quantifies the number of accessible electronic states within a given energy range. The x-axis of DOS graphs shows the energy levels of electrons in a molecule; higher energies are represented by positive values, while lower energies are represented by negative values. The LUMO is represented by the positive energy zone, and the HOMO is represented by the negative energy region. The relative abundance of electrons at each energy level is indicated by the y-axis. The graphic findings of the DOS analysis performed with the Multiwifn software^94,95^ are shown in Fig. 18. It is used to calculate the partial and total density of states of designed BAT/CMC/CuFe_2_O_4_/RhB. To evaluate the impact of the parent structure, it was divided into five fragments as shown in Fig. 18 for the partial density of states (PDOS). The total density of distribution displayed − 7.247184684 e V and is characterized by a lack of color. Fragment 1 showed − 7.190095169 e V for BAT/CMC/CuFe_2_O_4_, while Fragment 5 for the RhB atoms is -7.564986613 e V, indicating an interaction between them. In the ground state, the HOMO energy of BAT/CMC/CuFe_2_O_4_ is -12.73493 e V, which increases to -1.823164 e V upon excitation. The band gap between them and with the dye is -10.912 e V, indicating the distribution of RhB with the BAT/CMC/CuFe_2_O_4_ and a chemical interaction between them^96^.

## Conclusion

The photo-Fenton catalytic performance of metal-free and bimetallic catalysts was tracked by the decolorization of RhB dye in the attendance of solar radiation. Tests were conducted to determine how the process’s efficiency was affected by the concentration of dye, pH solution, and oxidant dosage. The optimum [H_2_O_2_] obtained was 90 mM, [RhB] was 40 mg/L, and pH was approximately 2.5, displaying degradation efficiencies of 41.5, 67.2, and 94.2% within 80 min of solar irradiation at room temperature. CuFe_2_O_4_ was superior to BAT in enhancing the photo-Fenton mineralization of RhB. Increasing the temperature promoted decolorization in the following order: BAT/CMC/CuFe_2_O_4_ > CuFe_2_O_4_ > BAT, revealing the endothermic characteristics of dye degradation. The outstanding performance can be ascribed to the activity of the amide-linkage polymer with cellulose composite and the CuFe_2_O_4_ moiety. This is due to the suitable band gap originating from the partially interrupted π-conjugation of electrons inside the cavity of BAT and the CONH bond, along with its hydrogen bond interaction with the -OH groups of cellulose. Applying distilled water, the investigation demonstrated the feasibility of recycling the produced catalysts for four cycles, resulting in a drop in degradation efficiency to 12% for BAT/CMC/CuFe_2_O_4_. Additionally, the interaction between BAT/CMC/CuFe_2_O_4_ and RhB dye was investigated by DFT, showing a chemical interaction due to the oxidation and reduction processes of Cu and Fe. In addition to hydrogen bonding interactions, π–π stacking and electrostatic forces conrribute to the stability of RhB dye on the catalyst surface.

## Supplementary Information

Below is the link to the electronic supplementary material.

Supplementary Material 1

Supplementary Material 2

## Data Availability

All data generated or analyzed during this study are included in this published article [and its supplementary information files].
