# Supplementary material for: Properties and computational insights of catalysts based on amide linked polymer for photo-Fenton remediation of Rhodamine B dye
Source: Sci Rep. 2025 Aug 20;15:30566. doi: 10.1038/s41598-025-13192-z (PMC12368231; doi:10.1038/s41598-025-13192-z)
Supplement: Supplementary file 1 — Supplementary Material 1 [file 41598_2025_13192_MOESM1_ESM.docx]

**Properties and Computational Insights of Catalysts Based on Amide Linked Polymer for Photo-Fenton Remediation of Rhodamine B Dye**

Asmaa M Fahim ^1^, and Khadiga Mohamed Abas ^2,^*

^1^Department of Green Chemistry, National Research Centre, Dokki, P.O. Box.12622, Cairo, Egypt

**Email:**asmaamahmoud8521@gmail.com, am.abdel-wahid@nrc.sci.eg

^2^ Physical Chemistry Department, Advanced Materials Technology and Mineral Resources Research Institute, National Research Centre, 33 El-Bohouth St., Giza 12622, Egypt **Email:**[mohamedkhadiga728@yahoo.com](mailto:mohamedkhadiga728@yahoo.com), [km.hassan@nrc.sci.eg](mailto:km.hassan@nrc.sci.eg) (K.M.Abas), Orcid: [0000-0003-2569-7484]

*Corresponding author. Khadiga Mohamed Abas. Tel: +201022197965. E-mail: [mohamedkhadiga728@yahoo.com](mailto:mohamedkhadiga728@yahoo.com); [km.hassan@nrc.sci.eg](mailto:km.hassan@nrc.sci.eg)

**Supporting information**

1. **Sonochemical Synthesis of Bimetal Oxide Nanoparticles**

CuCl_2_⋅2H_2_O (0.5 g) and FeCl_3_⋅6H_2_O (1.5 g) solutions were made in 30 milliliters of deionized water, followed by a 15-minute ultrasonication. After adding 20 ml of a 1 M NaOH solution dropwise, the ultrasonication process continued (frequency: 20 kHz, power: 70 W). The mixture was then processed through an ultrasonic homogenizer (SONOPULS HD 2070) with a maximum power of 70 W. Following the reaction, deionized water and alcohol were used to wash the solution multiple times. Additionally, this powder was dried for 12 hours at 80° C in an oven.

1. **Determination for Point of Zero Charge**





**Fig S1. Optimization of pH_PZC_ for evaluated catalysts.**
